# Detecting signals of seasonal influenza severity through age dynamics

**DOI:** 10.1186/s12879-015-1318-9

**Published:** 2015-12-29

**Authors:** Elizabeth C. Lee, Cécile Viboud, Lone Simonsen, Farid Khan, Shweta Bansal

**Affiliations:** Department of Biology, Georgetown University, Washington, District of Columbia USA; Fogarty International Center, National Institutes of Health, Bethesda, Maryland USA; Department of Global Health, George Washington University, Washington, District of Columbia USA; Department of Public Health, University of Copenhagen, Copenhagen, Denmark; IMS Health, Plymouth Meeting, Pennsylvania USA; Pfizer Inc., Collegeville, Pennsylvania, USA

**Keywords:** Influenza, Influenza-like illness, Severity, Metrics, Age patterns, Epidemiology, Mortality, United States

## Abstract

**Background:**

Measures of population-level influenza severity are important for public health planning, but estimates are often based on case-fatality and case-hospitalization risks, which require multiple data sources, are prone to surveillance biases, and are typically unavailable in the early stages of an outbreak. To address the limitations of traditional indicators, we propose a novel severity index based on influenza age dynamics estimated from routine physician diagnosis data that can be used retrospectively and for early warning.

**Methods:**

We developed a quantitative ‘ground truth’ severity benchmark that synthesizes multiple traditional severity indicators from publicly available influenza surveillance data in the United States. Observing that the age distribution of cases may signal severity early in an epidemic, we constructed novel retrospective and early warning severity indexes based on the relative risk of influenza-like illness (ILI) among working-age adults to that among school-aged children using weekly outpatient medical claims. We compared our relative risk-based indexes to the composite benchmark and estimated seasonal severity for flu seasons from 2001–02 to 2008–09 at the national and state levels.

**Results:**

The severity classifications made by the benchmark were not uniquely captured by any single contributing metric, including pneumonia and influenza mortality; the influenza epidemics of 2003–04 and 2007–08 were correctly identified as the most severe of the study period. The retrospective index was well correlated with the severity benchmark and correctly identified the two most severe seasons. The early warning index performance varied, but it projected 2007–08 as relatively severe 10 weeks prior to the epidemic peak. Influenza severity varied significantly among states within seasons, and four states were identified as possible early warning sentinels for national severity.

**Conclusions:**

Differences in age patterns of ILI may be used to characterize seasonal influenza severity in the United States in real-time and in a spatially resolved way. Future research on antigenic changes among circulating viruses, pre-existing immunity, and changing contact patterns may better elucidate the mechanisms underlying these indexes. Researchers and practitioners should consider the use of composite or ILI-based severity metrics in addition to traditional severity measures to inform epidemiological understanding and situational awareness in future seasonal outbreaks.

**Electronic supplementary material:**

The online version of this article (doi:10.1186/s12879-015-1318-9) contains supplementary material, which is available to authorized users.

## Background

The causes and characterization of population-level severity are crucial aspects to understanding influenza epidemiology and designing effective surveillance and control programs. Variation in seasonal influenza severity may be caused by environmental [1, 2], antigenic [3], strain-dependent [4], and epidemiological [5] factors, but this research has not been synthesized across fields and the mechanisms are not fully understood.

**Fig. 1:**
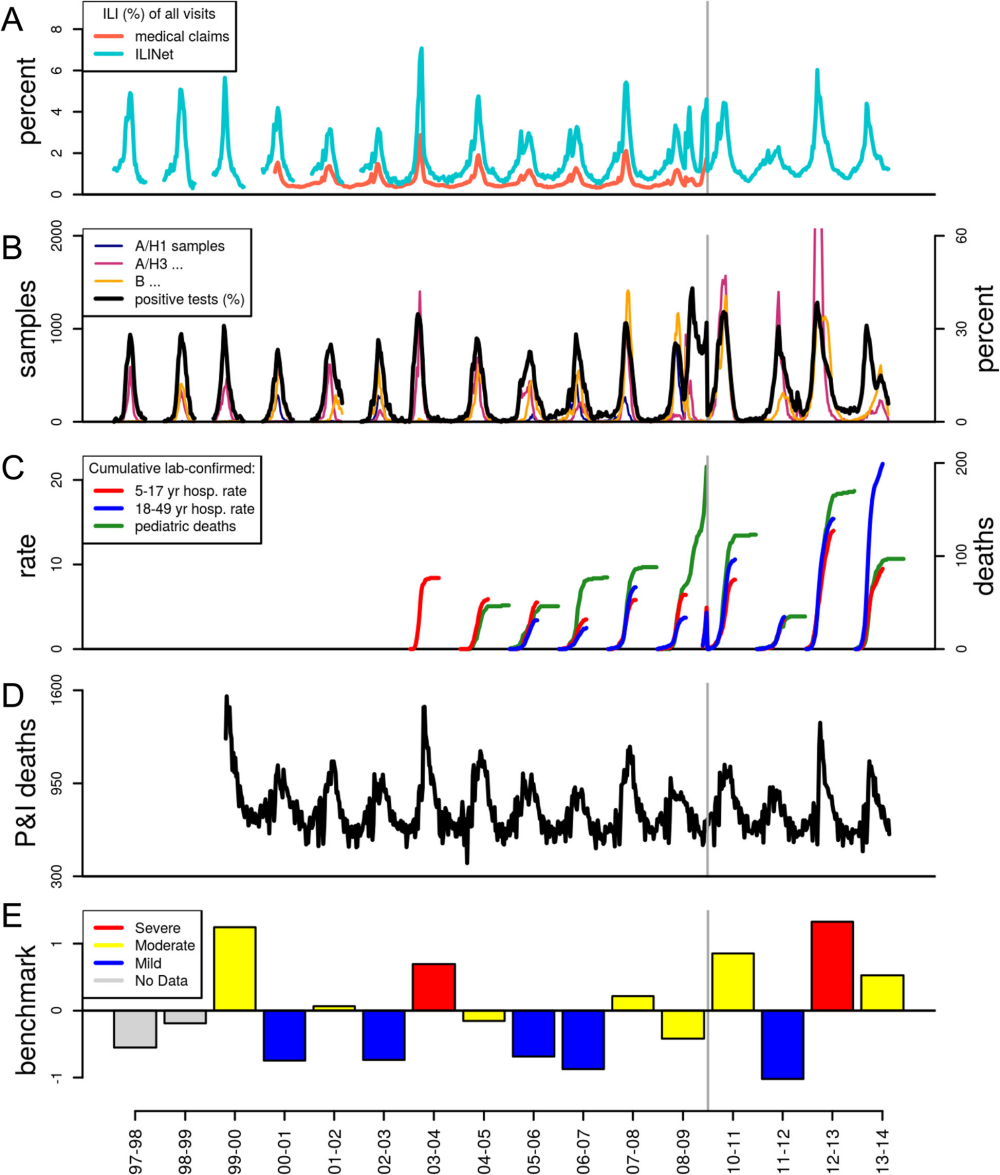
Influenza surveillance data in the United States for the 1997–98 to 2013–14 seasons (excluding 2009–10). Characterization of ILI activity as a function of: **a** ILI as a percentage of all outpatient visits in CDC’s ILINet and IMS Health medical claims data, **b** influenza subtype samples and percentage of laboratory-confirmed influenza specimens, **c** laboratory-confirmed influenza surveillance: cumulative hospitalization rates per 100,000 population for ages 5–17 and 18–49, and cumulative pediatric deaths (under 18 years old) over the course of the season, and **d** number of deaths attributed to pneumonia and influenza. The grey vertical line denotes a break in the time series for the period from October 2009 through September 2010; data not shown were not available. **e** The benchmark (*β*_*s*_) was constructed from surveillance data on positive percentage of influenza tests, hospitalization rates, pediatric deaths, and pneumonia and influenza deaths. Bar color corresponds to severity categories, qualitatively assigned in a textual analysis of CDC Flu Season Summaries

Current discourse about population-level seasonal influenza severity ties itself traditionally to experiences of severe patient-level outcomes. The United States Centers for Disease Control and Prevention (CDC) characterizes seasonal severity through influenza-associated hospitalization rates and mortality due to pneumonia and influenza (Fig. 1). From these surveillance data, CDC estimated a range of 3,000 to 49,000 influenza-associated all-cause deaths and over 200,000 hospitalizations per year in the United States during the period between 1976 and 2007 [6, 7]. Clinical studies similarly focus on patient-level outcomes, where physicians use scoring techniques to rate overall patient severity or the severity of specific symptoms [8, 9].

Many epidemiological analyses utilize aggregate measures of patient-level severity, such as case-fatality and case-hospitalization risk, to assess the severity of pandemic and emerging outbreaks [10–14]. Other studies model the relationship between excess mortality and morbidity rates [15, 16] or threshold excess pneumonia and influenza (P&I) mortality rates in order to identify and detect severe flu seasons. The CDC has recently adopted a population-level severity framework for influenza pandemics that incorporates both clinical severity and transmissibility metrics, but the clinical severity component remains closely tied to case-fatality and similar ratios [12]. These measures of severity based on mortality and hospitalization only capture one facet of the experience of flu across the population [4, 17], and are also limited by the availability of data. P&I mortality data are not collected in real-time by many national flu surveillance systems (e.g., in the European Union), and laboratory-confirmed hospitalization and mortality data that are collected are available with some delays (e.g., U.S. hospitalization data are backfilled due to data processing times) and for limited age groups (e.g., only laboratory-confirmed pediatric mortality is reported in the U.S.). Additionally, while hospitalization and mortality remain the accepted measures of influenza severity, there is no composite quantitative metric (used by the CDC or others) that synthesizes the varying acute effects imposed by the disease.

In this work, we develop novel severity assessment metrics that synthesize traditional severity measures on viral activity, hospitalizations, and deaths in the United States, and explore how the age patterns in influenza-like-illness (ILI) among the healthiest and largest segments of the population (children and working-age adults) may be used as proxies of population-level severity. Based on publicly-available epidemiological data, we first derive a composite benchmark that will serve as a quantitative ground truth for population-level influenza severity. Using a high coverage outpatient ILI dataset based on medical claims data from the United States, we then introduce two novel influenza severity metrics: 1) a retrospective index based on ILI age dynamics, which can aid in epidemiological analysis and the evaluation of public health responses using a commonly collected single data source; 2) an early warning index, estimated prior to the epidemic peak, which can help physicians improve patient-level communication, diagnosis, and treatment and inform decision makers on communication strategies regarding vaccination and antiviral usage.

## Methods

### Severity benchmark for each influenza season

We first created a synthetic composite *benchmark* for each season to represent a quantitative ‘gold standard’ indicator of severity. This benchmark integrated the following publicly available CDC surveillance data, aggregated to the flu season level: 1) percentage of influenzapositive laboratory confirmations among all tested respiratory specimens, 2) laboratory-confirmed influenza hospitalization rates among individuals five to seventeen years old and 3) eighteen to forty-nine years old, 4) number of laboratory-confirmed influenza deaths in children under 18 years, and 5) proportion of all deaths due to pneumonia and influenza (P&I) (time series displayed for the period from 1997–98 to 2013–14 in Fig. 1b-d). Data for items one through four may be accessed online through CDC’s FluView Interactive application [18]; data for item five may be accessed through the CDC WONDER Morbidity and Mortality Weekly Report web application [19]. Historically, CDC has used these surveillance sources to qualitatively consider multiple facets of influenza season severity. CDC’s outpatient ILI surveillance system ILINet (Fig. 1a), was another such historical source of severity, but it was excluded from the benchmark in order to prevent confounding when comparing the benchmark to an ILI-based severity index.

We generated a composite benchmark value for each flu season (*β*_*s*_, where *s* denotes the season) for the 16 seasons from 1997–98 to 2013–14 (excluding the 2009 H1N1 pandemic), which represented the entire period that CDC provided public surveillance data for flu. First, we performed a log transformation to the rate and count data streams (i.e., hospitalization rates, pediatric deaths) and a logit transformation to proportion and percent data (i.e., positive lab confirmations, P&I deaths) in order to put the various data types on the same scale. Second, we standardized each of these ‘raw’ metrics (*θ*_*i*,*s*_, where *i* denotes the data stream and *s* denotes the season) by the mean ($\mu _{\theta _{i}}$) and standard deviation ($\sigma _{\theta _{i}}$) across all available flu seasons, such that $\theta _{i, s}^{*} = (\theta _{i, s} - \mu _{\theta _{i}})/\sigma _{\theta _{i}}$, where * denotes the standardized metric. We took the mean of all available standardized raw metrics $\left (\theta _{i, s}^{*}\right)$ to generate the composite benchmark, *β*_*s*_, for a given season ($\beta _{s} = \left (\sum \limits _{i=1}^{n_{\theta }} \theta _{i, s}^{*}\right)/n_{\theta }$, where *n*_*θ*_ is the number of contributing data streams. Larger values of *β*_*s*_ indicate more severe seasons according to the benchmark, and vice versa.

Surveillance systems did not contribute to *β*_*s*_ when data were unavailable (Additional file 1: Table S2). Alternative standardization periods were considered in sensitivity analysis; the rank order of seasons according to *β*_*s*_ was mildly sensitive to these methodological changes and may be appropriate for severity assessment in different research contexts (Additional file 1: Figure S2).

For comparison, we determined categorical severity classifications (i.e., mild, moderate, severe) from a qualitative analysis of CDC influenza season summaries and Morbidity and Mortality Weekly Reports. This method is further described in Additional file 1: Section 3.1 and Additional file 2. These severity categories were used to provide additional context to Fig. 1e and Fig. 3.

### ILI medical claims data

The primary data for the remainder of our analysis comprised weekly physicians’ office and outpatient visits from October 2001 to May 2009 for influenza-like illness from a records-level database of CMS-1500 U.S. medical claims (Fig. 1a). This medical claims dataset incorporated 934 three-digit physician office U.S. zipcode prefixes (zip3s) and physician coverage increased from 22 to 70 % over the course of the study period; data were collected from 408,606 of 581,876 active physician practices during the 2008–09 flu season. We used a synthetic ILI indicator to represent influenza activity; this indicator was derived and validated in a previous study from a set of International Classification of Diseases, Ninth Revision (ICD-9) codes – influenza (487–488) or [fever and (respiratory symptoms or febrile viral illness) (780.6 and (462 or 786.2))] or prescription of oseltamivir (most commonly, 079.99) [20]. Recent analysis finds that ILI claims data accurately capture weekly fluctuations in influenza activity and season level intensity at high resolutions by age group and geographic location [20] and can be used to monitor the spatial spread of the disease [21]. See Additional file 1: SM section S1 for statements on ethics and data access. We considered the population of school-age children as 5–19 years old and working-age adults as 20–59 years old. Data were adjusted for differences in temporal coverage and age-specific care-seeking behavior. See Additional file 1: SM section S2 for further details on ILI data processing.

### Retrospective and early warning severity indexes based on ILI

Based on results from exploratory analysis (see Additional file 1: SM section S4), our first step towards developing a severity index was to calculate the relative risk (RR) of adjusted ILI (see Additional file 1: SM section S2) among adults to that in school-aged children: *RR*_*s*_(*t*)=*A*_*s*_(*t*)/*C*_*s*_(*t*), where *A*_*s*_(*t*) and *C*_*s*_(*t*) are the number of ILI cases captured in the surveillance system in a given week (*t*) in a season (*s*), divided by the group’s population size, in adults and school-age children, respectively (Additional file 1: Figure S6a). Since ILI is not restricted to laboratory-confirmed flu cases and baseline ILI activity varies from year to year, we standardized each season’s weekly RR time series (*rho*_*s*_(*t*)), such that $\rho _{s}(t) = \nicefrac {({RR}_{s}(t) - \mu _{{RR}_{s}})}{\sigma _{{RR}_{s}}}\phantom {\dot {i}\!}$, where $\mu _{{RR}_{s}}\phantom {\dot {i}\!}$ and $\sigma _{{RR}_{s}}\phantom {\dot {i}\!}$ were the mean and standard deviation of *RR*_*s*_(*t*) values during a specified *baseline period*. We defined this *baseline period* as the beginning of October to mid-November (weeks 40–46).

Two severity *classification periods* were identified under our framework, the *retrospective* (*r*) and *early warning* (*w*) periods. These periods were the only weeks *t* when *ρ*_*s*_(*t*) was significantly correlated with *β*_*s*_, which indicated the uniqueness of the signal detection during these periods. See Additional file 1: Section 5.1 and Additional file 1: Figure S4 for further detail on the identification of these periods. The retrospective period was the two week period that began three weeks before the ILI peak in a given flu season ILI curve; this period can only be identified *retrospective* to the epidemic peak. The early warning period was the two week period that began two weeks after the Thanksgiving holiday in the United States. We defined *retrospective severity* ($\overline {\rho _{s, r}}$) as the mean of *ρ*_*s*_(*t*) values during the retrospective period and *early warning severity* ($\overline {\rho _{s, w}}$) as the mean of *ρ*_*s*_(*t*) values during the early warning period.

Retrospective severity captured the disease dynamics of the primary epidemic growth period and could only be assessed after the epidemic peak had passed, while the early warning severity provided an earlier assessment of severity between the Thanksgiving and winter holidays. Severity was also reasonably well estimated with the age-specific ILI relative risk over the entire flu epidemic period, but use of the two-week retrospective period was preferred as it requires less data (Additional file 1: Figure S5). Early warning severity was not reported for early flu seasons (eg. 2003–04) because the early warning period coincided with the epidemic peak during this season. To compare *β*_*s*_ to $\overline {\rho _{s, r}}$ or $\overline {\rho _{s, w}}$, we calculated Pearson’s R correlation coefficients (*H*_*o*_:*R*=0) and reported *p*-values from a two-sided test of permutations without replacement. We also compared retrospective severity to traditional severity metrics, circulation of H3 strains, vaccine match and vaccine efficacy for seasons where these data were publicly available from CDC or reported in other studies [22] (Additional file 1: SM section S5).The primary analysis constructed and validated indexes developed from the medical claims ILI data, but a secondary analysis applied the same methods to construct relative-risk-based indexes from publicly available data from CDCŠs ILINet, and compared *β*_*s*_ to these relative-risk-based indexes, $\overline {\rho _{s, r}^{cdc}}$ and $\overline {\rho _{s, w}^{cdc}}$ (Additional file 1: SM section S8).

We assessed the sensitivity of the retrospective severity rank order to baseline period duration and found that the retrospective severity index was somewhat sensitive to changing baseline periods (Additional file 1: Figure S6b-d), but that our chosen period best represents baseline age dynamics (Additional file 1: Figure S7). We also performed analyses with ILI rates in excess of a seasonal baseline, and found that age dynamic patterns of relative risk remained similar for the medical claims data (Additional file 1: Figure S8).

### State-level analyses

To study regional patterns in influenza severity, we calculated relative-risk-based severity indexes for each U.S. state with the available, aggregated zip3-level data (See Additional file 1: SM section S6). State-level retrospective severity ($\overline {\rho _{s, r}(\tau)}$) and early warning severity ($\overline {\rho _{s, w}(\tau)}$), where states are represented as *τ*, were calculated with similar methods to national level indexes. The state-level retrospective period was tied to a state’s peak ILI week. (For example, in season *s*, California’s retrospective severity ($\overline {\rho _{s, r}(\tau)}$) is the two week period beginning three weeks before California’s peak ILI week). In these analyses, national retrospective and early warning indexes remain notated $\overline {\rho _{s, r}}$ and $\overline {\rho _{s, w}}$, respectively.

State-level retrospective severity was examined for each season. To identify states that may have had more severe or mild seasons relative to the rest of the United States, we calculated the state deviation from the national baseline as the relative difference between state and national retrospective indexes: ($(\overline {\rho _{s, r}(\tau)} - \overline {\rho _{s, r}})/|\overline {\rho _{s, r}}|$). To identify possible “sentinel” states for national influenza severity, we compared Pearson’s R correlation coefficients (*H*_*o*_:*R*=0) between state-level early warning ($\overline {\rho _{s, w}(\tau)}$) and national retrospective severity ($\overline {\rho _{s, r}}$) across seven study seasons (excludes 2003–04, where the early warning period occurred after the epidemic start). Tests across states were treated as independent, and *p*-values were calculated with a two-sided test of 1000 permutations without replacement.

## Results

### Severity benchmark

The composite severity benchmark (*β*_*s*_) identified 1997–98, 2000–01, 2002–03, 2005–06, 2006–07, and 2011–12 as the mildest seasons and 1999-00, 2003–04, 2010–11, and 2012–13 as the most severe seasons across the period from 1997–98 to 2013–14 (excludes the 2009–10 pandemic year) (Fig. 1e). While the peak percentage of influenza-positive test samples appeared higher among the most severe seasons, this data stream did not differentiate the mildest from the more moderate seasons (Fig. 1b). Laboratory-confirmed hospitalization rates and pediatric deaths varied across seasons, and only in more recent years (2010–14) did these measures appear to match benchmark severity magnitude (Fig. 1c). Peak P&I mortality was greatest among three of the four most severe seasons according to the benchmark, but the mildest and more moderate seasons had less clear separation.

Benchmark severity magnitude was not uniquely captured by any single contributing metric, supporting our use of a composite benchmark measure (Fig. 1b-d and Additional file 1: Figure S1). For example, the 2006–07 season was one of the mildest seasons according to the benchmark, and it had the lowest rates of child and adult hospitalization and P&I mortality compared to other seasons, but relatively high counts in pediatric deaths, suggesting that seasons could have mixed indications of severity across different data streams. More severe seasons like 1999-00, 2003–04, and 2012–13 tended to have high P&I mortality at the peak, but they did not necessarily have a greater percentage of influenza-positive laboratory tests. Moreover, high P&I mortality was not a sufficient condition to indicate severity, as demonstrated by the severe 2010–11 season. In comparing the data across seasons, the benchmark integrated these indicators into a single quantitative value that captured the magnitude of these multiple facets of severity.

### Measuring severity through age-specific illness risks

**Fig. 2:**
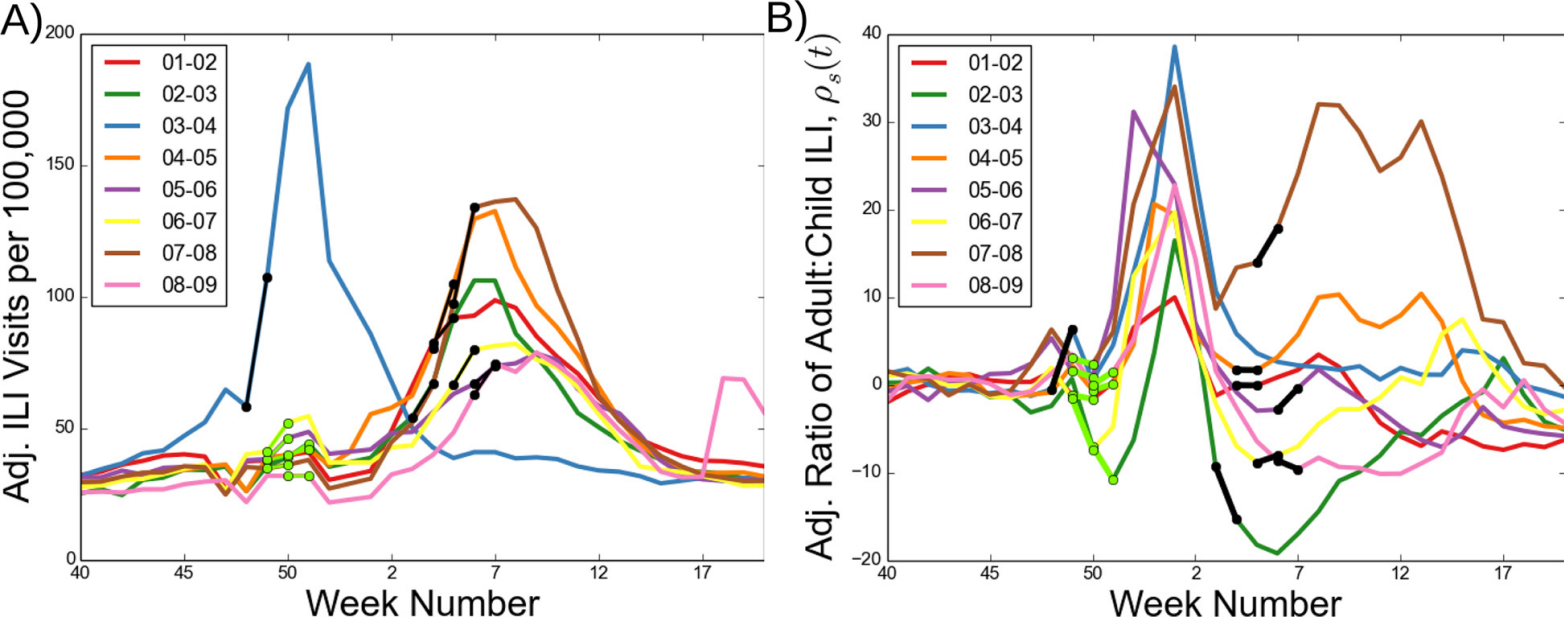
Influenza age dynamics differ from overall epidemic dynamics. **a** Medically attended outpatient ILI visits per 100,000 for the 2001–02 through 2008–09 flu seasons, adjusted for increasing surveillance data coverage and ILI care-seeking behavior, are displayed. The national early warning and retrospective classification periods are overlaid in green and black, respectively. **b** The normalized relative risk of adult ILI to child ILI rates (*ρ*_*s*_(*t*)), a proxy of age-specific disease burden, follows a regular seasonal pattern during the U.S. Thanksgiving and winter holiday periods, and diverges during the typical epidemic growth periods of January and February (around weeks 2–7)

We were motivated to study ILI age patterns for epidemiological and empirical reasons. While elderly and young child populations are considered high-risk for severe influenza outcomes and are the traditional source of direct measurements of influenza severity, we adopted an indirect approach by considering ILI rates in high transmission age groups: adults and children. Children are thought to play an important role in influenza transmission due to high numbers of contacts [23, 24], while working-age adults represent a large part of the population, bridge contact between age groups, and have greater within-group contact heterogeneity [24, 25]. We operationalized this relationship by using weekly ILI data (Fig. 2a) to consider a weekly proxy of age-specific disease burden, *ρ*_*s*_(*t*), which is a standardized relative risk of adult to child ILI rates at week *t* (Fig. 2b). We emphasize that our metric is not a proxy for seasonal transmissibility; rather, it is formulated from the relative age distribution of cases.

**Fig. 3:**
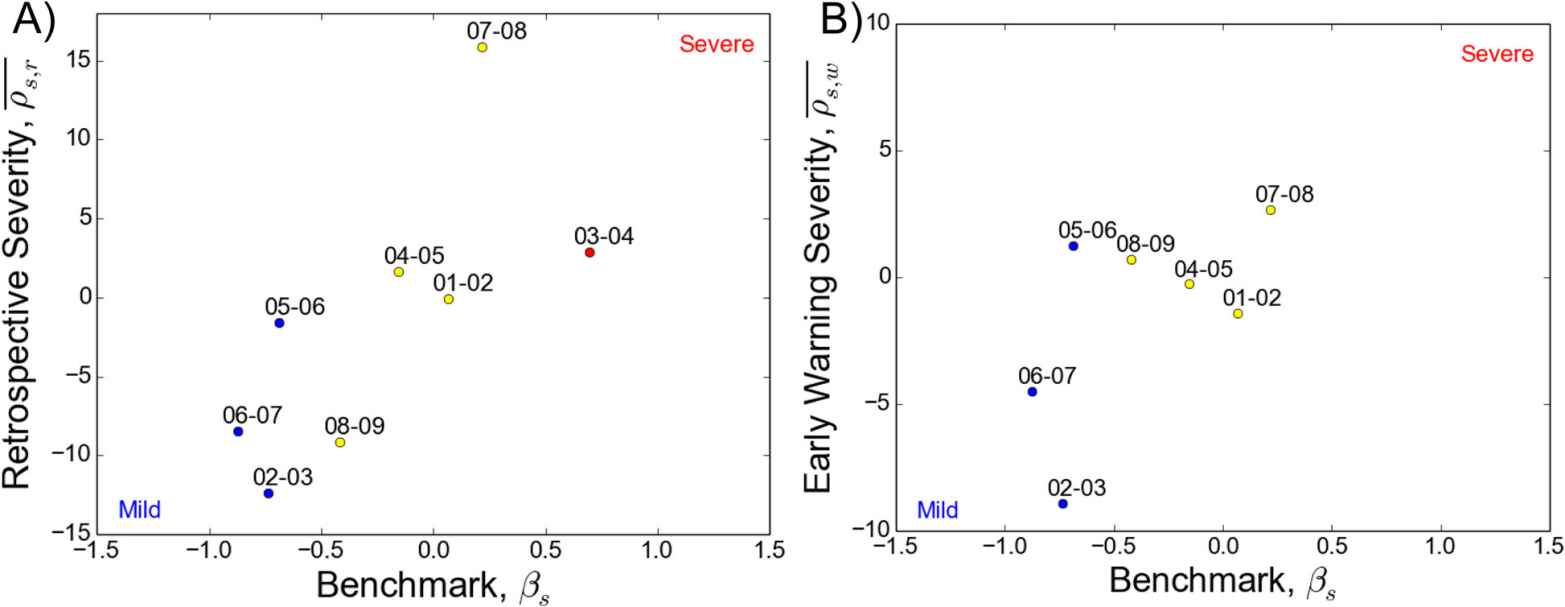
Retrospective and early warning severity indexes compared to the benchmark. **a** Retrospective severity ($\overline {\rho _{s, r}}$) has a positive relationship with the benchmark (*R*= 0.71, *p*-value = 0.05). **b** Early warning severity ($\overline {\rho _{s, w}}$) has a positive relationship with the benchmark (*R*= 0.59, *p*-value = 0.16). The 2003–04 season was removed because it was an early flu season and the early warning period occurred after the retrospective period. Point color corresponds to qualitatively-assigned severity category, where *red* is severe, *yellow* is moderate, and *blue* is mild

We compared our relative risk-based severity measures (retrospective $\overline {\rho _{s, r}}$ and early warning $\overline {\rho _{s, w}}$) with quantitative classifications such as the benchmark (*β*_*s*_) and other traditional severity metrics. During the 2001–02 to 2008–09 study period, retrospective severity ($\overline {\rho _{s, r}}$) identified 2002–03, 2006–07, and 2008–09 as the mildest seasons and 2003–04 and 2007–08 as the most severe seasons. Retrospective severity was moderately correlated with the benchmark (Pearson’s R = 0.71, *p*-value =0.05 when compared to *β*_*s*_ classifications) (Fig. 3a). The early warning index ($\overline {\rho _{s, w}}$) projected 2007–08 as relatively severe and 2002–03 and 2006–07 as relatively mild; the correlation was weaker with the benchmark (Pearson’s R = 0.59, *p*-value = 0.16) (Fig. 3b). Note that the 2003–04 season was removed from this analysis because it peaked during the early warning period (Fig. 2a). Among traditional severity metrics, total season ILI visits also had a positive relationship with retrospective severity $\overline {\rho _{s, r}}$ (Additional file 1: Figure S9). Proportion of H3 subtype circulation had a weak positive relationship (Additional file 1: Figure S10) while a proxy of vaccine match had a negative relationship with retrospective severity $\overline {\rho _{s, r}}$ (Additional file 1: Figure S11).

Next, we repeated this analysis where ILINet, the traditional ILI surveillance system maintained by the CDC, was used instead of medical claims data to calculate the relative risk severity indexes. The early warning index $\left (\overline {\rho _{s, w}^{cdc}}\right)$ did not appear to have a linear relationship with *β*_*s*_. Nevertheless, we found that the retrospective index $\left (\overline {\rho _{s, r}^{cdc}}\right)$ had a strong positive relationship with *β*_*s*_ (Pearson’s R = 0.64, *p*-value = 0.01) (Additional file 1: Figure S13), and that the retrospective indexes for ILINet and the medical claims had a strong positive relationship to each other (Pearson’s R = 0.78, *p*-value = 0.02) (Additional file 1: Figure S14).

### State-level severity patterns and sentinels

**Fig. 4:**
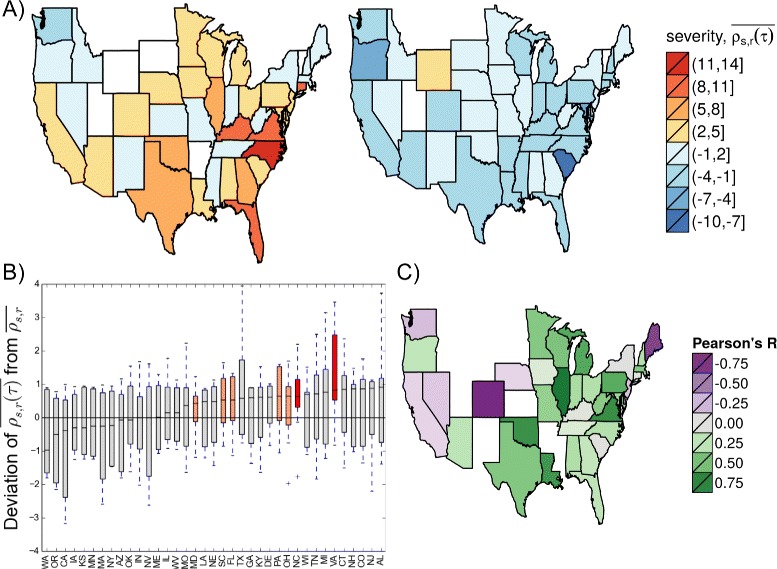
State-level patterns of seasonal influenza severity. **a** State retrospective severity ($\overline {\rho _{s, r}(\tau)}$) may range from mild to severe in a single season regardless of the national retrospective severity index ($\overline {\rho _{s, r}}$). The 2007–08 (*left*) and 2008–09 (*right*) seasons, where $\overline {\rho _{s, r}}$ values were 16 and -9 respectively, are displayed. States in white did not have sufficient data to calculate a retrospective severity index. **b** Deviation between state ($\overline {\rho _{s, r}(\tau)}$) and national retrospective severity ($\overline {\rho _{s, r}}$) across the eight study seasons was used to identify states that tend to experience more severe flu seasons than other states. The 75^*th*^ and 70^*th*^ percentiles exceeded zero for red and orange highlighted states, respectively. **c** Pearson’s R correlation coefficients (*H*_*o*_:*R*=0) between state early warning ($\overline {\rho _{s, w}(\tau)}$) and national retrospective ($\overline {\rho _{s, r}}$) classifications were used to suggest possible ‘sentinel’ states. Only coefficients for Illinois, Virginia, Colorado and Maine had *p*-values below 0.05. States in white did not have enough data to calculate at least one of the two metrics for at least one study season

We examined spatial severity patterns by calculating retrospective and early warning indexes from age-specific ILI rates at the state-level (based on the medical claims data). Regardless of national retrospective severity ($\overline {\rho _{s, r}}$), state-level retrospective severity ($\overline {\rho _{s, r}(\tau)}$) could range from mild to severe in a single season (Fig. 4a). Across the eight study seasons, the adjacent Mid-Atlantic states of Virginia and North Carolina may have experienced more severe seasons than national $\overline {\rho _{s, r}}$ (75^*th*^ percentile of state deviation was above zero), and other adjacent Mid-Atlantic and Midwestern states like Ohio, Pennsylvania, Florida, South Carolina, and Maryland may have experienced somewhat more severe seasons (70^*th*^ percentile of state deviation was above zero) (Fig. 4b). No state was highlighted for experiencing milder flu seasons than the rest of the U.S., but western states had the lowest median $\overline {\rho _{s, r}(\tau)}$ indexes across the study period (Additional file 1: Figure S12).

In a separate analysis, we explored whether “sentinel” states, where early warning ($\overline {\rho _{s, w}(\tau)}$) was strongly correlated with national retrospective severity ($\overline {\rho _{s, r}}$), could be identified. In Fig. 4c, we examined correlation coefficients between $\overline {\rho _{s, w}(\tau)}$ and $\overline {\rho _{s, r}}$ among the 36 states with data available for the seven study seasons (excludes the early 2003–04 season). Illinois and Virginia had early warning indexes $\overline {\rho _{s, w}(\tau)}$ with strong positive correlations with $\overline {\rho _{s, r}}$ (Pearson’s R = 0.82, 0.72; *p*-values = 0.01, 0.04, respectively), while Colorado and Maine had a strong negative correlation with $\overline {\rho _{s, r}}$ (Pearson’s R =−0.80, −0.71, *p*-value =0.02, 0.03, respectively).

## Discussion

In this study, we have developed a composite indicator that synthesizes different influenza data streams to provide a quantitative benchmark of seasonal influenza severity. We have also developed a novel severity index based on age-related patterns of influenza-like illness that can be used in both retrospective and early warning contexts. Motivated by our finding that adult ILI visits were highly correlated with traditional measures of severity like hospitalization and deaths, we developed a proxy for influenza severity based on the ratio of ILI risk among adults relative to that among children. As school-aged children and adults are at the lowest risk for seasonal influenza complications and death [26], our metric seeks to measure signals of severity indirectly through populations that are well-represented in influenza case data and well-connected to high-risk populations. The retrospective severity index had a positive correlation with the benchmark, while the early warning index tended to err conservatively from the standpoint of public health (i.e., early warning signals predicted more severe seasons than occurred).

We constructed the composite severity benchmark to synthesize publicly available influenza surveillance data in the United States, and have shown that it agrees with epidemiological understanding of historical CDC reports of past influenza seasons. The benchmark thus captures multiple facets of severity in composite form and fills a gap in the current literature where quantitative ground truth measures of population-level influenza severity are absent. With additional data availability, future applications of the benchmark may add weights to contributing data streams or apply alternative normalization methods according to researcher or practitioner needs. Despite its contribution to public health, this measure remains limited by its contributing data sources: these data streams are not available in real-time, their data collection methods and definitions may change substantially across seasons, and they are not readily collected at different spatial scales or in different countries.

**Fig. 5:**
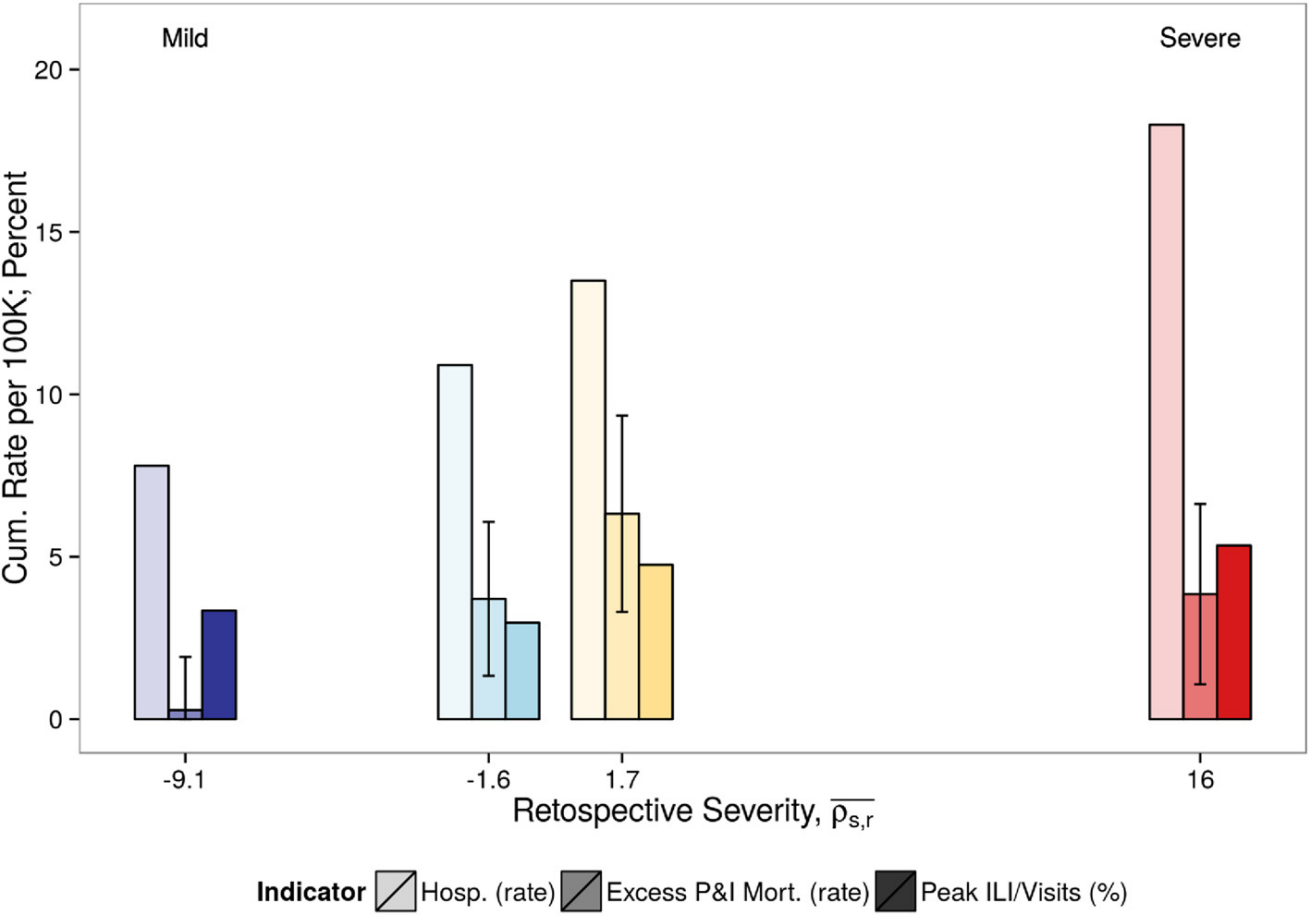
Translation of retrospective severity to operational indicators of the burden of influenza. The retrospective severity index ($\overline {\rho _{s, r}}$) may be mapped to historical data on cumulative confirmed influenza-related hospitalizations per 100,000, peak week outpatient visits due to ILI (ILINet), and seasonal excess P&I mortality rates per 100,000 in order to inform decision makers about the expected range of disease burden in a given season. Error bars represent the standard deviation in state-level variation of the excess P&I mortality rate, and bar color represents a milder to more severe retrospective severity index value (*dark blue* to *dark red*)

Our novel relative risk-based severity indexes based on ILI age patterns aim to address the limitations of traditional severity measures. The retrospective index may inform public health systems evaluations and enable historical analysis of severe season attributes, which will improve our understanding of influenza disease ecology. In relation to existing severity measures, this index can be used with a single data stream, and a source of data (i.e., ILI) that is commonly collected in routine influenza surveillance in many countries and at local departments of health. The performances of our early warning index remain modest, perhaps owing to the limited number of seasons available for study. In theory, however, this or an improved early warning index, determined 9–12 weeks before the typical epidemic peak, may enable clinicians to make informed decisions about patient diagnosis and treatment strategies, and help hospitals to plan staffing and supply logistics during an outbreak. Individual health-related behaviors may change during an epidemic as a result of health communication campaigns regarding pharmaceutical [27] and behavioral [28, 29] interventions; in pursuit of these goals, the early warning index presents a novel attempt at real-time severity estimation. To make the use of our metrics more intuitive and to provide an example of how they may be used in an operational context, we map the retrospective severity index to functional indicators of influenza burden, including peak ILI, hospitalization, and mortality rate in Fig. 5. (See Additional file 1: SM section S7 for the calculation of operational indicators).

The extension of our index to state-level patterns highlights how scalable severity metrics have the potential to improve the observation of broader epidemiological trends and forge new directions (e.g., spatial signals of early warning) to inform public health preparedness. The low data requirements of the relative risk-based indexes enable continued future study over longer time periods, which may help elucidate the mechanisms that drive spatial variation in severity within individual seasons. Additional validation of state-level severity indexes is needed, but the future identification of robust state sentinels could improve multi-scale planning and coordination efforts months before resources are widely demanded.

Instead of focusing on the elderly and young children as traditional high-risk groups [30], our retrospective and early warning indexes look for indirect signals of severity using the disease dynamics of ‘healthier’ populations. Measurement of ILI among high-risk groups at outpatient facilities may be unreliable, as those groups may be seeking care at hospitals for severe pathology. Instead, we use the more reliable signals provided by measurement of ILI among working adults and school-age children. We posit that school-aged children experience substantial flu morbidity every season because they have high numbers of potential disease-causing contacts [24, 31, 32] and greater susceptibility due to limited prior exposure to influenza. We hypothesize that adults have fewer contacts and greater prior exposure than children, so they experience high flu activity only when the flu season is severe, regardless if the cause is strain novelty, higher transmissibility, greater virulence, or some combination of factors. High connectivity between adults and other age groups [24] and the role of adults in seeding new regions [33, 34] may underlie our observation that seasons with high burden in adult populations tend to be severe for the entire population. In demonstrating the potential of this metric, we call for the continued collection of age-specific ILI data and additional research on the development of thresholds to define and differentiate mild from severe seasons.

Further work is needed to improve severity index signal detection in the early warning period, and extra caution should be taken when making decisions based on the early warning index. This period sometimes experiences low influenza circulation, thus allowing pathogens like respiratory syncytial virus (RSV) and *Haemophilus influenzae* to confound the ILI age dynamics used in our index [35]; however, we note that low circulation is rare in our study period (Additional file 1: Table S3). Additionally, the fixed nature of the early warning period limits its utility for early-peaking flu seasons (e.g., 2003–04). Future research should explore methods to represent uncertainty in severity assessments; action upon incorrect predictions could lead to overburdening the health care system or the inefficient use of resources, and a mismatch in expectations and reality could result in a loss of public trust in public health agencies. Moreover, the early warning index for ILINet surveillance did not perform well; this may be explained by ILINet’s smaller sample size (roughly 1,900 providers submitted weekly reports in 2013–14 to ILINet, while over 400,000 physicians reported to the medical claims data in 2008–09) and narrower syndromic definition of flu compared to the medical claims data, both of which could limit the detection and classification of influenza activity during the early warning period (Additional file 1: Figure S13). Nevertheless, our observations of ILI age dynamics in this early warning period (around weeks 49–52) lead us to hypothesize that the predictable age dynamic shifts in the abutting Thanksgiving and winter holidays, which may be due to reduced contact rates, create an insulated ‘severity testbed’ for improved signal detection during these weeks. Future research on holiday age dynamics and early flu seasons with different ILI surveillance systems may in fact reveal that the early warning index is limited to use in the United States.

The relative risk-based severity indexes are limited in their detection capabilities for influenza pandemics. Pandemic events are characterized by different distributions of age risk, which may alter the severity classifications provided by our index; an initial pandemic wave may be dominated by morbidity among school-aged children, and empirical and modeling studies suggest that adults are more likely to become infected in the season following a pandemic [5, 36–39]. Moreover, there appears to be an accumulation of heterosubtypic immunity for pandemic strains with age [40]. Our index would not capture severity in the first and second waves of pandemic virus circulation, which is why we exclude the 2009–10 season from our analysis, and unstable age dynamics in post-pandemic seasons may explain poor performance of recent seasons in the ILINet analyses (Additional file 1: Figure S13c-d).

Our novel severity index relies on real-time age-specific medical claims data for ILI, which does not appear to have the disadvantages of flu-related ‘big data’ sources [20, 41]. Traditional ILI surveillance (eg. ILINet) also provides real-time age-specific data, but the medical claims database represents a more obligatory form of provider reporting, captures ILI activity at least as well as traditional surveillance, and provides higher coverage, greater spatial resolution, and finer age-specific disease information due to its administrative purpose [20]. Medical claims and ILINet data are both subject to physician biases regarding the demographics and seasonality of influenza and doctor’s office closures. They also have healthcare-seeking behavior biases; school-aged children have higher rates of healthcare-seeking behavior for ILI than adults (approximately 1.1 to 1.4 times higher) [42–44], which we consider in the construction of our index (See Additional file 1: SM section S2). Additional studies on disparities in insurance and access to care, especially in consideration of ongoing changes to the U.S. health care system, are needed to better quantify biases in medical claims data as compared to other flu surveillance systems.

## Conclusions

Traditional measures of seasonal influenza severity are limited by their need for multiple data streams and the lack of accurate hospitalization and mortality data in real-time. In our study, relative disease burden among adults and children is proposed as the basis for a novel population-level severity index with retrospective and early warning classification periods, and the index is applied to influenza-like illness data in the United States across multiple seasons and spatial scales. By correctly identifying the two most severe influenza seasons in the study period, this work represents proof of concept that influenza age dynamics may provide epidemiological understanding beyond surveillance data at face value, and our approach may be used by physicians, hospital administrators, and policy makers to make real-time decisions about clinical, logistical, and strategic responses to a seasonal influenza outbreak. While further study of the novel severity metrics is warranted, we recommend that researchers and practitioners consider the use of composite or ILI-based metrics in addition to traditional severity measures for improved epidemiological understanding and situational awareness. Our research raises new questions about causal severity mechanisms; future analyses should disambiguate the age patterns characterized in our study as a harbinger or result of population-level severity, examine the hypothesis that holiday contacts seed broader infection in different age groups [45] or new locations, and examine different regional subtype circulation, pre-existing immunity, age distributions, or vaccine coverage rates as mechanisms for spatial variation in severity.

## Additional files

Additional file 1
**Supporting material (SM).** Detailed descriptions about data processing, methodological choices, sensitivity analyses, and supporting evidence. The Supporting Material (SM) content includes sections S1 to S8, Tables S1 to S3, and Figures S1 to S14. (PDF 2212 kb)

Additional file 2
**Historical CDC documentation of past influenza seasons.** Table of qualitatively-assigned severity categories and surveillance system text descriptions drawn from Centers for Disease Control and Prevention (CDC) influenza season summaries and Morbidity and Mortality Weekly Reports (MMWR) for the 1997–98 through 2013–14 flu seasons. Severity categories were assigned according to the algorithm described in Additional file 1: Supporting material section S3.1. (XLSX 23 kb)

## Abbreviations

CDC: United States Centers for Disease Control and Prevention
ICD-9: International Classification of Diseases, Ninth Revision
ILI: influenza-like illness
MMWR: Morbidity and Mortality Weekly Reports
P&I: pneumonia and influenza
RR: relative risk
RSV: respiratory syncytial virus
