# Supplementary material for: Detecting signals of seasonal influenza severity through age dynamics
Source: BMC Infect Dis. 2015 Dec 29;15:587. doi: 10.1186/s12879-015-1318-9 (PMC4696185; doi:10.1186/s12879-015-1318-9)
Supplement: Additional file 1 — Supporting material (SM). Detailed descriptions about data processing, methodological choices, sensitivity analyses, and supporting evidence. The Supporting Material (SM) content includes sections S1 to S8, Tables S1 to S3, and Figures S1 to S14. (PDF 2212 kb) [file 12879_2015_1318_MOESM1_ESM.pdf]

Detecting signals of seasonal influenza severity through age dynamics: supporting material

Contents

S1 Ethics and data access 1

S2 ILI data processing 1

S3 Severity benchmark 1
S3.1 Qualitative severity analysis for benchmark 'ground-truthing' . . . 2
S3.2 Sensitivity analyses for the benchmark normalization period . . . 3

S4 Factors associated with influenza severity 4

S5 Retrospective and early warning severity indexes 4
S5.1 Identifying the retrospective and early warning periods . . . 4
S5.2 Sensitivity analysis for the severity index baseline period . . . 5
S5.3 Sensitivity analysis for the severity index with excess ILI data . . . 6
S5.4 Comparison of severity index to traditional severity measures . . . 6
S5.5 Comparison of severity index to H3 circulation . . . 7
S5.6 Comparison of severity index to vaccine match and effectiveness . . . 7
S5.7 Influenza circulation during the early warning period . . . 7

S6 State-level analyses 8
S6.1 Data processing . . . 8
S6.2 State-level severity patterns . . . 9

S7 Operational public health indicators 9

S8 Applying the severity index to ILINet surveillance data 9
S8.1 CDC ILINet data . . . 10
S8.2 Severity index for CDC's ILINet data . . . 10

S1 Ethics and data access

Patient records and information in the medical claims dataset were anonymized, de-identified, and aggregated by IMS Health, a data analytics firm that packages data that is routinely collected for health insurance purposes for commercial use. Mr. Farid Khan, Director of Advanced Analytics at IMS Health, granted the researchers access to the data. While the database is not accessible online, interested researchers should refer to the IMS Health website: http://www.imshealth.com/portal/site/imshealth. All analyses were performed with aggregated time series data for influenza-like illness rather than patient-level information. Similar to other epidemiological analyses of administrative insurance data, no institutional review board approval was sought.

S2 ILI data processing

We adjusted ILI cases to account for increasing coverage over time and spatial and age-specific estimates of health-care seeking behavior for ILI in the medical claims data. Thus, the adjusted data represented all ILI for the population covered within the medical claims data in the last year of our study period (2008-09). For example, estimates of true, weekly ILI visit rate in season s for region n covered in the 2008-09 flu season was calculated as:

$$Tr_{s,g,n}(t) = ili_{s,g,n}(t) * (v_{S08-09,n}/v_{s,n})/cs_{g,n},$$

where $Tr_{s,g,n}(t)$ represents the true influenza-like illness in week t of season s, population g (i.e., total population, children, or adults), and region n (i.e., entire U.S. or specific state) in terms of the data coverage in 2008-09. $ili_{s,g,n}(t)$ is influenza-like illness observed in the data, $v_{s,n}$ is the total number of outpatient visits recorded by the data during season s, and $cs_{g,n}$ estimates health care-seeking behavior for ILI [1, 2].

The total population care-seeking estimate was population weighted by 2010 Census child and adult population estimates and weighted by survey size across the September 2009 to March 2010 and January to April 2011 Behavioral Risk Factor Surveillance System (BRFSS) surveys conducted by CDC. National age-specific care-seeking estimates were survey size-weighted averages across the two studies. State-level age-specific care-seeking estimates were taken only from the September 2009 to March 2010 BRFSS; where survey data was unavailable for a specific state, the age-specific Census region estimate was used (Table S1) [1]. Adjusted ILI visit rate per population was used in all reported analyses.

Table S1: Summary of estimates of percent that sought care for ILI by age group and spatial scale (national or U.S. Census region) according to BRFSS surveys. The national estimate was used to adjust total incidence for care-seeking and Census region estimates were used to adjust state-level incidence when state-specific estimates (not shown) were unavailable from the 2009-10 BRFSS. Estimates were unable for adults in Vermont, and children in Arkansas, Colorado, North Carolina, and Vermont. These adjustments account for possible differences in care-seeking behavior across age groups, which could affect the classifications of the severity index.

Sought Care for ILI (%)	National	Northeast	Midwest	South	West
Total Pop	45	47	41	48	37
Children	51	58	48	66	50
Adults	41	44	39	42	33

S3 Severity benchmark

A synthetic composite severity benchmark (β_s) was developed for the 1997-98 through 2013-14 flu seasons (excluding 2009-10). We used the benchmark to identify factors associated with severity and to compare a 'gold standard' quantitative measure of severity to the retrospective and early warning severity indexes. Data sources described in Table S2 were aggregated to create β_s and unavailable data were excluded from the composite measure. Almost all of the raw CDC surveillance data had a strong positive relationship with the benchmark, suggesting that there is no unique indicator of season-level severity among these surveillance

systems (Figure S1). For greater robustness and in acknowledgment of multiple causes of season-level severity, we used a composite measure of these CDC surveillance sources as the benchmark.

Since the 2009 H1N1 pandemic, testing practices in the United States have changed, including the more frequent use of rapid influenza-diagnostic tests and reverse-transcription polymerase chain reaction (RT-PCR) tests and the increased practice of pre-screening respiratory specimens by public health laboratories [3]. Consequently, the percent of positive lab confirmations may not serve as a valid indicator of seasonal influenza activity in a β_s that includes influenza seasons occurring after 2009.

Table S2: CDC surveillance data sources contributing to the severity benchmark (β_s), by season. *Positive Isolates* is the percentage of positive lab confirmations among all specimens tested. *P&I Deaths* is the proportion of all deaths that are associated with pneumonia and influenza. *Pediatric Deaths* is the number of flu-associated deaths in children less than 18 years old, which is a nationally notifiable condition in the US. *Hospitalizations* (5-17 and 18-49 years) is the sum of the laboratory-confirmed hospitalization rate per 100,000 individuals in the population among states participating in EIP. Seasons beginning 2005-06 incorporate all five metrics into the severity benchmark.

Metric ($\theta_{i,s}$)	97-98	98-99	99-00	00-01	01-02	02-03	03-04	04-05
Positive Isolates[a]	x	x	x	x	x	x	x	x
P&I Deaths[b]			x	x	x	x	x	x
Pediatric Deaths[c]								x
Hospitalizations (5-17 years)[d]							x	x
Hospitalizations (18-49 years)[d]								

[a] Source: WHO & NREVSS Collaborating Laboratories
[b] Source: 122 Cities Mortality Reporting System
[c] Source: Influenza Associated Pediatric Mortality
[d] Source: Emerging Infections Program (EIP)

S3.1 Qualitative severity analysis for benchmark 'ground-truthing'

To ground truth benchmark assessments to historical consensus opinion of seasonal influenza severity, we performed a qualitative analysis of influenza season summaries and Morbidity and Mortality Weekly Reports (MMWRs) published by CDC for 14 of the 16 seasons for which a benchmark value was calculated (See Additional file 2). The texts describing the 1997-98 and 1998-99 seasons did not report enough data on prior influenza seasons for the qualitative analysis. See Additional file 2 for the source texts and codes by season.

We applied the following algorithm to determine the severity classification (mild, moderate, severe) of each flu season. First, we assumed that the four seasons (2000-01, 2002-03, 2003-04, 2012-13) described with the specific terms 'mild' or 'moderately severe' were mild or severe seasons, respectively ('moderately severe' was the most severe qualitative description for any season). Second, we analyzed the available descriptions that corresponded to the CDC 'factors contributing to severity' —percentage of positive isolates at the peak, number of consecutive weeks with elevated positive isolate activity, percentage of P&I deaths or patient visits due to ILI at the peak, number of consecutive weeks that P&I deaths or ILI visits exceeded the epidemic threshold, number of states reporting regional or widespread influenza activity at the peak, number of pediatric deaths, and cumulative laboratory-confirmed hospitalization rates among children and adults during the flu season. Not all metrics were available for each season. To classify the remaining 10 seasons, we tallied the number of reported metrics that fell below, within, or above the range of CDC-reported values for the previous two to four flu seasons (as available in the text). In an objective assignation, if at least 50% of reported measures were below this range, the season was 'mild'; if at least 50% of reported measures were above this range, the season was classified 'severe'; all other seasons were classified 'moderate'.

Using this algorithm, 2003-04 and 2012-13 were classified as severe, 1999-2000, 2001-02, 2004-05, 2007-08, 2008-09, 2010-11, and 2013-14 were moderate, and 2000-01, 2002-03, 2005-06, 2006-07, and 2011-12 were mild (Figure 1e, Additional file 2). We compared the rank order of the benchmark values to the clustered severity categories and reported the number of rank order discrepancies (e.g., the number of times that a season with a 'severe' qualitative classification had a quantitative benchmark value below a season classified as 'moderate').

Figure S1: Benchmark vs. original CDC surveillance data comprising benchmark (all available seasons during 2001-02 to 2008-09 period are shown): A) hospitalization rate among individuals 18-49 years old per 100,000, B) hospitalization rate among individuals 5-17 years old per 100,000, C) number of pediatric deaths, D) percentage of all-cause mortality due to P&I (Pearson's R = 0.84), and E) percentage of positive flu isolates confirmed (Pearson's R = 0.91). Correlation coefficients are reported only for metrics where data were available for all seasons in the eight season study period. Point color corresponds to qualitatively-assigned severity category, where red is severe, yellow is moderate, and blue is mild.

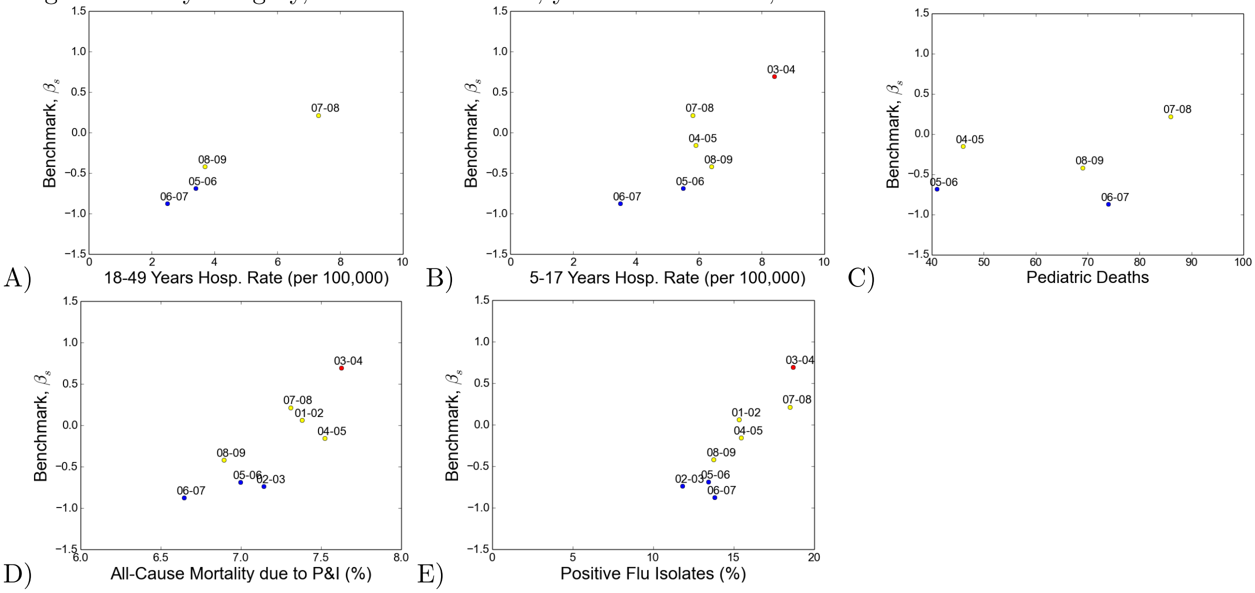

There was good agreement between benchmark rank orders and qualitatively-assigned severity categories. Two discrepancies between the benchmark and qualitative severity category were identified; the 1999-2000 and 2010-11 flu seasons were classified as 'moderate' in the qualitative analysis, but their benchmark values are greater than that of the 2003-04 flu season, which was classified as 'severe' in the qualitative analysis (Figure 1e).

S3.2 Sensitivity analyses for the benchmark normalization period

We performed sensitivity analyses on the period across which raw factors are normalized. In the results presented in the main text, we standardized each raw metric $\theta_{i,s}$ across the complete period with available data (1997-98 to 2013-14) and compared this to the retrospective severity index $\overline{\rho_{s,r}}$ (Figure S2a). Given the antigenic changes to circulating viruses over this period, we compared the classifications of this grouping to those of two other standardization periods: 1) 1997-98 to 2002-03, 2003-04 to 2008-09, and 2010-11 to 2013-14, which represents three periods with similar antigenic clusters [4] (Figure S2b), and 2) 1997-98 to 2008-09 and 2010-11 to 2013-14, which represents the pre- and post-2009 H1N1 pandemic period (Figure S2c).

Rank order of seasons according to the benchmark (β_s) was mildly sensitive to changes in the grouping scheme; 2006-07 and 2002-03 swapped places when comparing the complete period grouping (Figure S2a) to the pre-/post-pandemic grouping (Figure S2c). Standardization across these alternative periods may better identify the mildest and most severe seasons within each period, but they may also complicate comparisons of seasons in different periods. In future work, alternative periods may be chosen according to their appropriateness to the research question.

Figure S2: Alternative benchmark (β_s) normalization schemes for comparison to the retrospective severity index ($\overline{\rho_{s,r}}$). A) Complete period grouping (same as Figure 3A in main text): 1997-98 to 2013-14 (excluding 2009-10) (Pearson's R = 0.71). B) Antigenic cluster grouping: 1997-98 to 2002-03, 2003-04 to 2008-09, and 2010-11 to 2013-14 (Pearson's R = 0.76). C) Pre-pandemic and post-pandemic grouping: 1997-98 to 2008-09 and 2010-11 to 2013-14 (Pearson's R = 0.77). Point color corresponds to qualitatively-assigned severity category, where red is severe, yellow is moderate, and blue is mild.

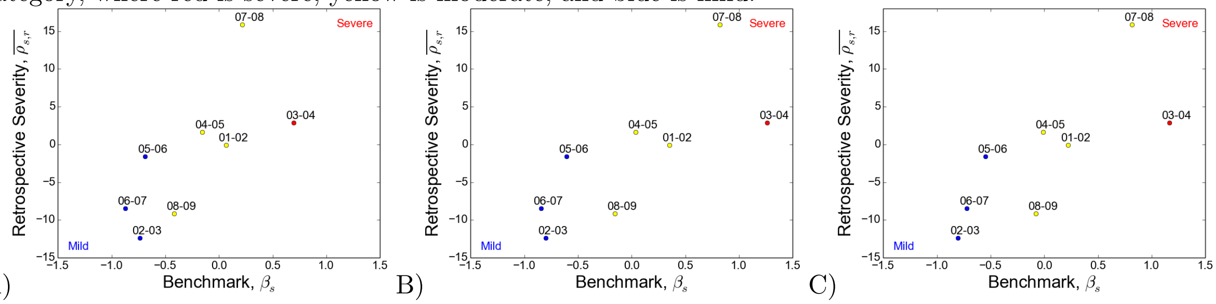

S4 Factors associated with influenza severity

We conducted an exploratory analysis to identify possible drivers of seasonal influenza severity. For the 1997-98 to 2013-14 flu seasons (excluding 2009-10), we examined pairwise Pearson cross-correlation coefficients of the severity benchmark (β_s) with the following nation-level covariates ($H_o : R = 0$): 1) the proportion of all positive influenza laboratory samples characterized as H3 at the end of the season, as reported by World Health Organization (WHO) and National Respiratory and Enteric Virus Surveillance System (NREVSS) laboratories, 2) seasonal ILI visit rates among toddlers aged 0-4, 3) children aged 5-24, 4) adults aged 25-64, 5) elderly aged 65 and above, and 6) the total population, as reported by ILINet, and average flu season 7) precipitation and 8) temperature for 48 contiguous U.S. states from NOAA's National Data Climate Center (NCDC). P-values were calculated with a two-sided test of 1000 permutations without replacement, and pairwise tests of association between β_s and covariates of interest were deemed significant if they achieved p-value ≤ 0.05.

These covariates were chosen for their purported relationship with seasonal influenza severity in the literature. Previous studies suggest that H3 seasons may be more severe [5], and cold and dry conditions have been thought to favor influenza spread [6, 7]. Total attack rate was included because it is a simple measure of flu season magnitude. ILI visit rates in other age groups were included with the following motivations: children are thought to be primary local transmitters of disease due to high numbers of contacts [8, 9] and likely lower pre-existing immunity, toddlers and the elderly are considered high-risk groups for influenza-associated mortality due to weaker immune systems [10], and adults comprise the largest part of the population, bridge contact between age groups, and have great within-group heterogeneity in age-related contact patterns [9, 11].

Using the *corrplot* package in the *R* statistical programming language [12], we found that seasonal ILI visit rates in adults (R = 0.68, p-value < 0.001) and in the total population (R = 0.51, p-value = 0.03) were the only covariates with significant correlations with the benchmark (Figure S3).

S5 Retrospective and early warning severity indexes

S5.1 Identifying the retrospective and early warning periods

We performed an analysis to identify specific periods of the flu season that were highly correlated with the benchmark (Figure S4). For every overlapping two week moving average of $\rho_s(t)$ (where this moving average was denoted $\overline{\rho_s(t)}$, we computed Pearson's R correlation coefficients between the vector β_s and 1000 permutations of the seasonal order of vector $\overline{\rho_s(t)}$ (i.e., drawing without replacement) ($H_o : R = 0$, two-sided test). The 95% confidence interval corresponded to the 2.5 and 97.5 percentiles of those randomized correlation coefficients at each window period. Weeks 1 to 6 and window week period 49-50, which corresponded roughly

Figure S3: A) Visualization of the cross-correlation matrix where numbers represent the Pearson's R cross-correlation coefficients. Ellipse shape and color denotes the strength and direction of the correlation coefficient. The following covariates were compared to the benchmark: proportion of circulating H3 strains (H3), seasonal ILI visit rate among toddlers 0-4 (ili_t), children 5-24 (ili_c), adults 25-64 (ili_a), elderly 65 and above (ili_e), and the total population (ili_tot), and average flu season precipitation (precip) and temperature (temp) in the United States. B) Visualization of the cross-correlation matrix, where an 'X' marks correlations that are not significant according to a two-sided test of association of Pearson's R.

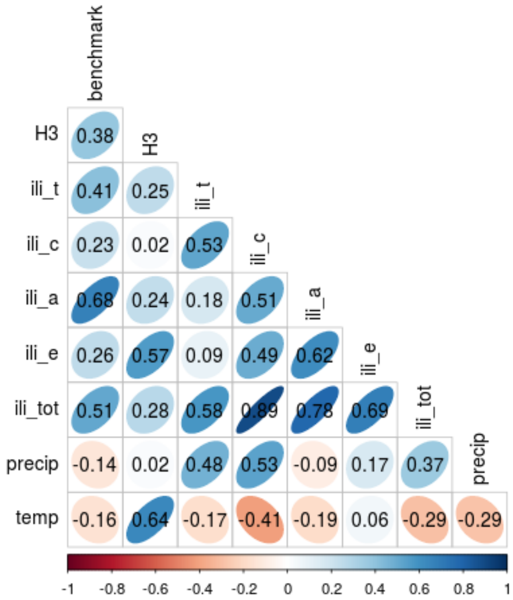

A)

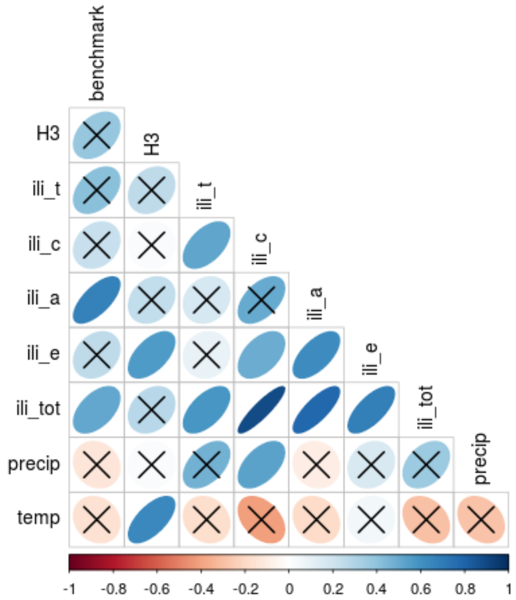

B)

to the retrospective and early warning periods across all seasons, were significantly above the 95% confidence interval of correlation between $\overline{\rho_s(t)}$ and β_s. These results suggested that age dynamics during these two fleeting periods provided strong signals of severity.

In addition to using the retrospective and early warning periods, severity may be estimated at the end of each flu season by looking at the aggregated RR over the entire flu epidemic period ($RR_{s,epi}$), which we defined as week 50 to week 12 (Figure S5a). We also examined the aggregated RR during the non-epidemic period ($RR_{s,nonepi}$), defined as weeks 21 to 39, in order to assess whether the correlations between $RR_{s,epi}$ and β_s were spurious (Figure S5b). The aggregated $RR_{s,epi}$ had a correlation of $R = 0.83$ ($H_o : R = 0$, two-sided Pearson's R permutation test, p-value = 0.01) with β_s, while RR_{nonepi} had a correlation of $R = 0.22$ (p-value = 0.61), thus confirming our expectation that only flu season age dynamics provide a signal of severity. While the entire epidemic period provided a strong signal of severity, the values were not as easy to interpret as those of ($\overline{\rho_{s,r}}$). For this reason, use of $\overline{\rho_{s,r}}$ for the retrospective index was preferred.

S5.2 Sensitivity analysis for the severity index baseline period

The *baseline period* presented in the main text was the first seven weeks of the potential flu season, the beginning of October to mid-November (weeks 40-46). We chose this pre-season baseline because it overlapped neither with the typical peak flu season in the United States, nor with the two classification periods for our severity indexes. We assessed the sensitivity of $\overline{\rho_{s,r}}$ to definitions of the baseline periods that were longer (ie. weeks 40-49) and earlier (ie. the summer period, weeks 33-39). We reasoned that the unpatterned variation in $RR_s(t)$ during the summer prior to the flu season start was graphically comparable to the variation in the baseline period (Figure S6a), and that seasonal differences may be captured at baseline during this time.

We found that rank order retrospective severity was quite consistent between the seven and ten week fall baselines, with a single swap between 2006-07 and 2008-09 (Figure 3a, Figure S6b). Both summer baselines had multiple discrepancies compared to the the seven week fall baseline rank order (Figure S6c-d), but the

Figure S4: For every two week period starting with weeks 40 and 41 (window period 4041), we examined the correlation coefficient between the moving average of $\rho_s(t)$, denoted $\overline{\rho_s(t)}$, and β_s across flu seasons in the study period. The red lines indicate the 95% confidence interval range for the correlation between β_s and mean $\rho_s(t)$, had the correlations been random. The windows corresponding to the early warning and retrospective periods were the only correlations that achieved statistical significance.

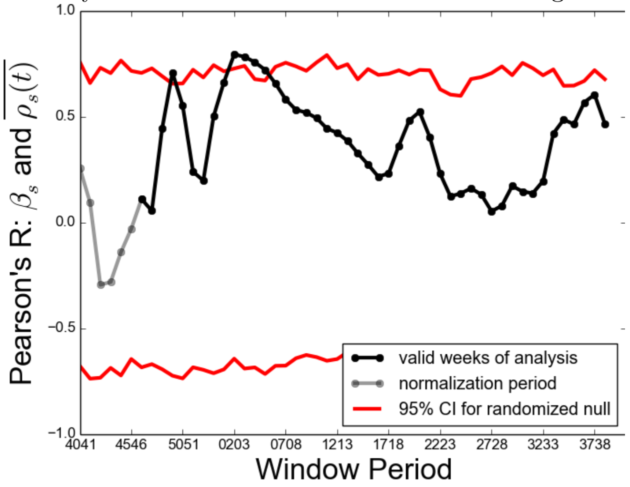

positive relationship across seasons remained consistent across all four baseline periods. The summer $RR_s(t)$ baselines may have had greater variation than the fall baselines due to fewer overall insurance claims reports across all diagnoses (Figure S7), which further justified the use of the week 40-46 fall seasonal baseline over summer baselines. Among fall periods, we preferred the seven week baseline period over the ten week period for analyses in the main text because less information was required and correlation strength with β_s was similar.

S5.3 Sensitivity analysis for the severity index with excess ILI data

Due to concerns that medical claims data captured too much non-flu ILI activity during the early warning period, we examined the patterns of relative disease burden among adults and children with excess ILI data. We calculated excess ILI from medical claims data ILI by subtracting an age-specific baseline constant of ILI activity from adult and child ILI visits. For each age group, the baseline constant was computed as the mean ILI visit rate over summer weeks (weeks 21 to 39) for the past two consecutive years plus two standard deviations, which is a slight modification of CDC's baseline calculation for their outpatient ILI network, ILINet.[1]

We found that using excess ILI reduced the magnitude of the ratio between adult and child ILI, but did not change the overall population or age dynamics of the results (Figure S8). Use of excess ILI did, however, complicate the calculation of the relative risk. The period of flu season that exceeded the ILI baseline was not consecutive, leading to the possibility of negative severity index values and difficulty in interpretation. For this reason, use of the processed 'regular' ILI data was preferred.

S5.4 Comparison of severity index to traditional severity measures

We compared retrospective severity ($\overline{\rho_{s,r}}$) to traditional severity measures derived from CDC surveillance data (sources described in 'Severity benchmark' sections) such as hospitalization rate, multiple proxies of risk of case fatality risk, and total seasonal ILI visit rates, and inpatient ILI visit rates from the medical claims data (Figure S9). There were positive relationships between $\overline{\rho_{s,r}}$ and hospitalization rates, the ratio of P&I deaths to ILI, and seasonal ILI rates (Pearson's R = 0.74), but relationships were less strong among

[1] Accessed at http://www.cdc.gov/flu/weekly/overview.htm on February 12, 2015.

Figure S5: A) Relative risk of adult to child ILI visits over the course of the entire epidemic flu period, defined as weeks 50 to 12 in a given season, was well correlated with the benchmark ($R = 0.83$, p-value = 0.01), while B) the non-epidemic, summer period did not appear to provide a signal of severity ($R = 0.22$, p-value = 0.61). Point color corresponds to qualitatively-assigned severity category, where red is severe, yellow is moderate, and blue is mild.

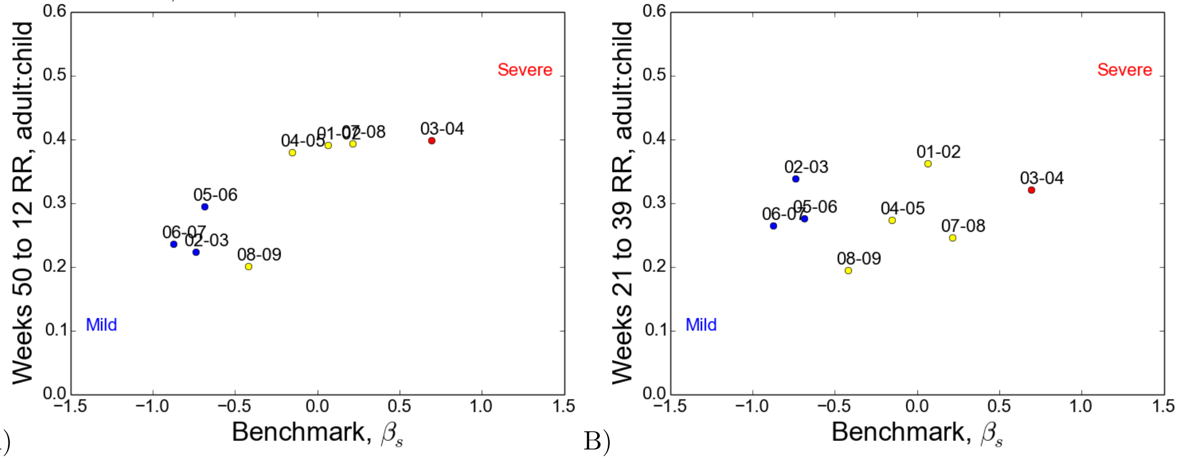

other measures. In conjunction with Figure 5, these figures might enable comparison of severity index values to more familiar severity measures.

S5.5 Comparison of severity index to H3 circulation

We explored the relationship between the retrospective severity index and the percentage of H3 strains circulating in a given season, as reported by the CDC virological surveillance system NREVSS. (Figure S10). We found that more severe seasons tended to have higher proportions of H3, but our severity index had the capacity to capture relatively severe non-H3 seasons.

S5.6 Comparison of severity index to vaccine match and effectiveness

We compared retrospective severity to seasonal vaccine attributes, such as trivalent vaccine match and vaccine efficacy. In their influenza season summaries, CDC reported the number of characterized influenza viruses that were antigenically similar to the corresponding subtype component in the seasonal vaccine. We estimated trivalent vaccine match by calculating the percentage of characterized viruses that matched a strain in the seasonal vaccine. We note that this method of estimating vaccine match does not capture the interactive effect of the strains in the vaccine. Vaccine efficacy percentages were calculated as the weighted average of study size across trivalent and live-attenuated influenza vaccine (LAIV) efficacy trials reported in other studies [13].

We did not include vaccine match and vaccine efficacy in our cross-correlation exploratory analysis because data were not available for all seasons from 1997-98 through 2013-14. Instead, we performed this graphical comparison between vaccine-related proxies and retrospective severity (Figure S11). We found some evidence to suggest that better vaccine match between circulating and vaccine strains (Pearson's R = -0.80). Vaccine match was confounded with H1 subtype seasons, because vaccine strains tended to match more closely during H1 seasons, and H1-dominant seasons tended to be more mild. Vaccine coverage estimates were examined because data were not available for the period of interest.

S5.7 Influenza circulation during the early warning period

We examined the mean percentage of positive laboratory confirmed isolates of influenza according to the National Respiratory and Enteric Virus Surveillance System (NREVSS) during the early warning period, which occurred during weeks 49-50 or weeks 50-51 each year. The mean percentage of positive isolates ranged

Figure S6: A) Differences in $RR(t)$ at the beginning of different flu seasons motivated our decision to normalize $RR(t)$ to make it comparable across seasons. Consequently, was constructed our age-specific disease burden proxy ($\rho_s(t)$) as $RR_s(t)$ that had been standardized by the baseline period. We compared the benchmark to retrospective severity ($\overline{\rho_{s,r}}$) where $\rho_s(t)$ had been calculated with alternative baseline periods of B) 10 weeks in the fall (weeks 40-49; Pearson's R = 0.75), C) 7 weeks in the preceding summer (weeks 33-39; Pearson's R = 0.67), and D) 10 weeks in the preceding summer (weeks 30-39; Pearson's R = 0.70). The 7 week fall baseline period was presented in our main text analyses. Summer baselines and longer baselines shifted the value of $\overline{\rho_{s,r}}$ towards the center. Point color in B-D corresponds to qualitatively-assigned severity category, where red is severe, yellow is moderate, and blue is mild.

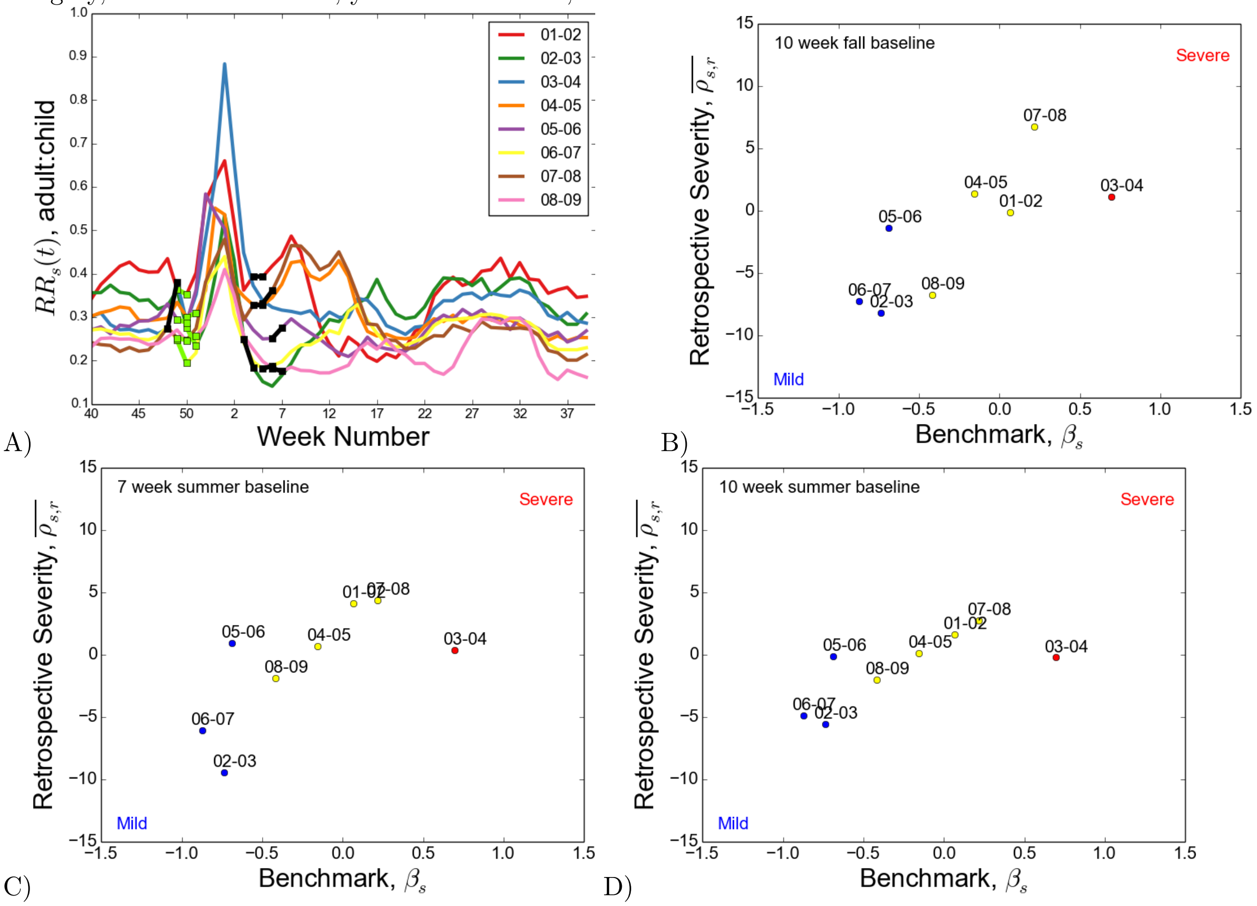

from 2% to 32% over the 1997-98 to 2013-14 flu seasons (excluding 2009-10) during the early warning period S3.

S6 State-level analyses

S6.1 Data processing

Almost all zip3s were contained within a single state's boundaries, and two zip3s that were located in multiple states were assigned to the primary state listed by the US Postal Service 3-digit zip code prefix matrix. [2] Zip3 ILI data and population data were aggregated to the state-level before generating weekly incidence rates.

[2]Accessed at http://pe.usps.com/text/LabelingLists/L002.htm on August 22, 2014.

Figure S7: Any diagnosis visits were reported during the seven week baseline periods during the fall and summer. The fall baseline period typically had a greater number of visits than the summer period. Since the fall baseline period was more proximate to epidemic flu season and had a greater number of overall visits, it was more suitable than summer as a baseline period for removing the effects of non-flu age dynamics during the flu season.

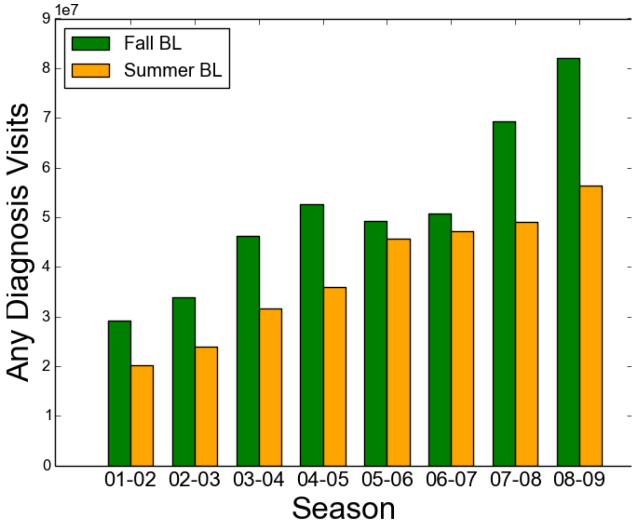

S6.2 State-level severity patterns

We plotted the empirical distribution of retrospective severity indexes across seasons by state. Across the 2001-02 to 2008-09 study period, we showed that a group of western states (CA, OR, WA, AZ) had the mildest median severity (Figure S12). States with the greatest absolute severity appeared to line the Atlantic coast.

S7 Operational public health indicators

In Figure 5, we compared a sample of relative risk-based severity index values to commonly used influenza public health indicators, which may better situate decision makers in known flu season contexts. Percent of national peak week outpatient visits due to ILI was identified using CDC ILINet data, and *peak week* achieved the greatest number of ILI cases in a single week during a given season. National cumulative hospitalization rate was obtained from the same public CDC data as that included in the benchmark.

We calculated state-level excess P&I mortality rates by state and season over the 2001-02 to 2008-09 study period from monthly pneumonia and influenza deaths at all places of death. These data were provided by the CDC WONDER Multiple Cause of Death database, which is publicly available online. As in Viboud et al. 2005, non-flu P&I mortality, which excludes the flu season period from December to April, was fit with a Serfling-type regression model to identify the seasonal baseline. December to April P&I mortality in excess of the baseline were categorized as excess P&I mortality, and aggregated to the season level [14]. National excess P&I mortality rates by season represented the population-weighted average of all state-level excess P&I mortality rates in that season.

S8 Applying the severity index to ILINet surveillance data

We applied our novel severity index framework to CDC's ILINet, a publicly available U.S. ILI surveillance dataset, in order to examine the applicability of our proposed metric to different data sources.

Figure S8: Excess ILI for weekly medical claims data with the national early warning and retrospective classification periods in green and black, respectively (Analog of Figure 2A in the main text). Negative values are below the baseline. Medically attended outpatient excess ILI visits per 100,000 is similar to overall ILI visits for the 2001-02 through 2008-09 flu seasons.

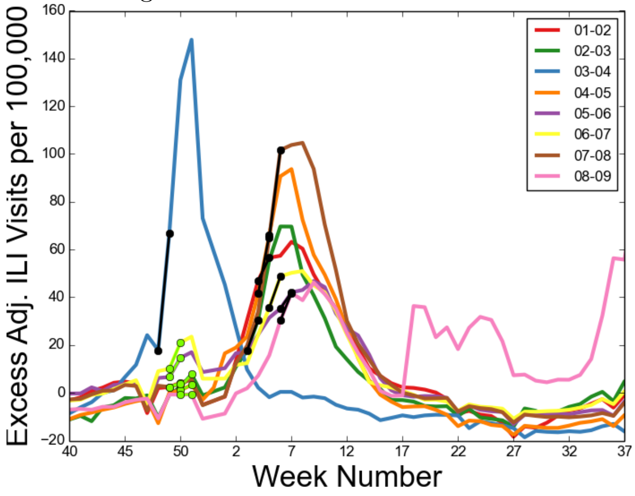

S8.1 CDC ILINet data

We compared our results to outpatient ILI data from CDC's ILINet, which reports surveillance data from approximately 1,800 health care providers in the U.S. each week, includes a total of over 2,900 providers in the entire system, and is available from October 1997 to May 2014. By 2014, the data documented outpatient visits from approximately 1,900 health care providers in the U.S. each week. [3] Similar to the medical claims data, ILINet defines ILI as [fever (100°F [37.8°C] or greater) and (sore throat or cough)] without a known cause other than influenza. These data were also available at the weekly level, across HHS regions, and by age group. To calculate the relative risk of ILI between adults and chidlren, school-age children comprised individuals aged 5-24 and adults comprised individuals aged 25-64. Data were available from October 1997 to May 2014, but they were not reported during the summer in the early years. As with the medical claims data, ILINet data were adjusted for age-specific care-seeking rates and total number of patient visits, as described in section S2.

S8.2 Severity index for CDC's ILINet data

Similar to the methods applied to the medical claims data, we calculated the retrospective and early warning severity indexes for the ILINet ILI visit time series data (Figure S13a) from the standardized weekly relative risk, $\rho_s^{cdc}(t)$ (Figure S13b). The retrospective period was the two week period that began three weeks prior to peak ILI as reported by ILINet in a given season. See Methods for additional details.

We compared the ILINet-based retrospective ($\overline{\rho_{s,r}^{cdc}}$) and early warning ($\overline{\rho_{s,w}^{cdc}}$) severity indexes to the benchmark (β_s). We examined data over the period from 1997-98 to 2013-14, excluding the 2009-10 season due to 2009 H1N1 pandemic. There was a positive relationship between $\overline{\rho_{s,r}^{cdc}}$ and β_s ($R = 0.64$, $H_o : R = 0$, two-sided Pearson's R permutation test, p-value = 0.01), but the early warning index ($\overline{\rho_{s,w}^{cdc}}$) was not correlated with β_s (R = -0.17, p-value = 0.59) (Figure S13c-d). The poor performance of both indexes for post-2009 seasons may be due to empirical changes in age dynamics in this period [15]. Absent post-pandemic seasons, a stronger positive linear relationship would have likely been observed between $\overline{\rho_{s,w}^{cdc}}$ and β_s.

ILINet retrospective severity ($\overline{\rho_{s,r}^{cdc}}$) had a tendency to underestimate severity as compared to the medical claims ($\overline{\rho_{s,r}}$) (Figure S14a), and there appeared to be a non-linear relationship between early warning indexes. Additional applications of the RR-based index to different ILI data sources are needed to better understand these sensitivities.

[3]See CDC overview of ILINet surveillance system: http://www.cdc.gov/flu/weekly/overview.htm. Accessed on 7/28/14.

Table S3: Mean percentage of positive laboratory confirmed isolates of influenza according to NREVSS in early warning period weeks from the 1997-98 to 2013-14 flu seasons, excluding 2009-10.

Season	Positive (%)
1997-98	5.2
1998-99	1.8
1999-00	21.2
2000-01	6.3
2001-02	4.1
2002-03	5.2
2003-04	31.9
2004-05	5.8
2005-06	7.1
2006-07	6.3
2007-08	3.9
2008-09	3.7
2010-11	16.1
2011-12	2.5
2012-13	31.4
2013-14	24.4

Figure S9: The retrospective severity index compared to other traditional severity measures (all available seasons during 2001-02 to 2008-09 period are shown): A) Hospitalization rate is the lab-confirmed flu hospitalization per 100,000 individuals in the population from CDC's Emerging Infections Program. B) The ratio of P&I mortality risk and ILI case proportion was derived from two CDC surveillance systems. The numerator is the proportion of all deaths attributed to P&I from the 122 Cities Mortality Reporting System. The denominator is the proportion of all visits due to ILI from ILINet. C) Cumulative number of P&I deaths from the 122 Cities Mortality Reporting System was divided by the cumulative number of ILI visits to physicians as reported by ILINet. D) Inpatient ILI visit rate from the medical claims data, where the service location was an inpatient/acute care facility or the emergency room (Pearson's R = 0.44). E) Seasonal ILI visit rate from the medical claims was well correlated with the retrospective severity index (Pearson's R = 0.74). ILI visit rates were adjusted for coverage and health care-seeking for ILI. Correlation coefficients are reported only for metrics where data were available for all seasons in the eight season study period. Point color corresponds to qualitatively-assigned severity category, where red is severe, yellow is moderate, and blue is mild.

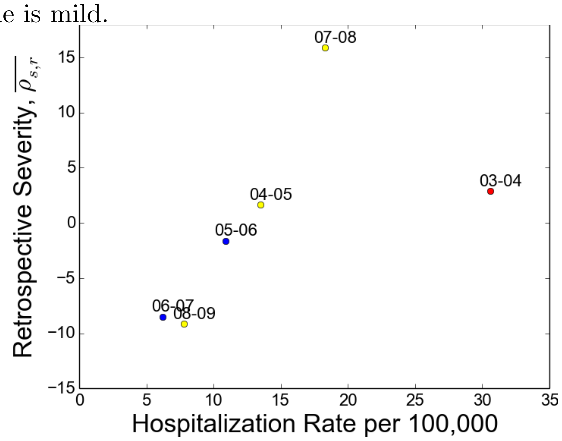

A)

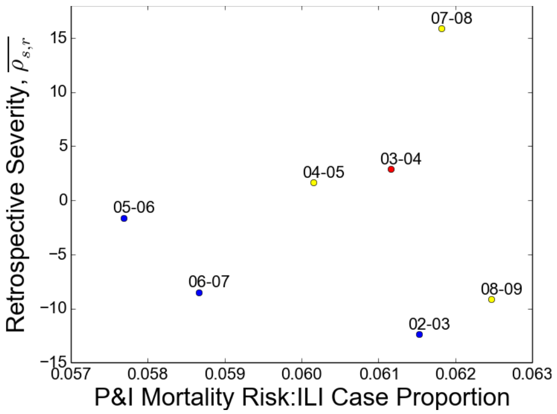

B)

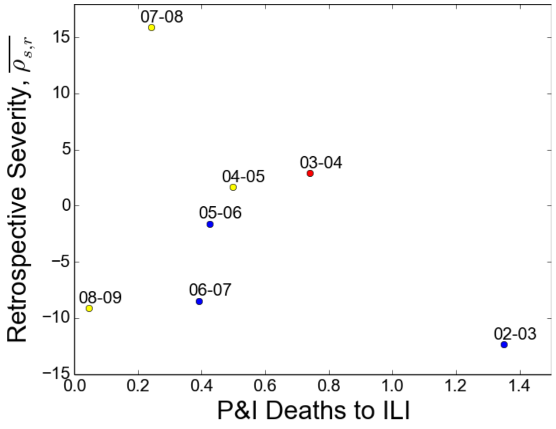

C)

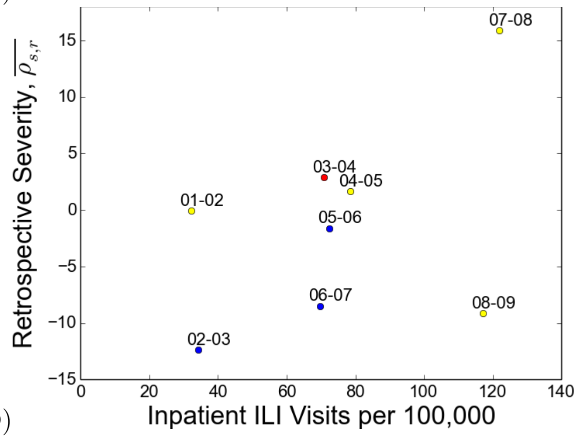

D)

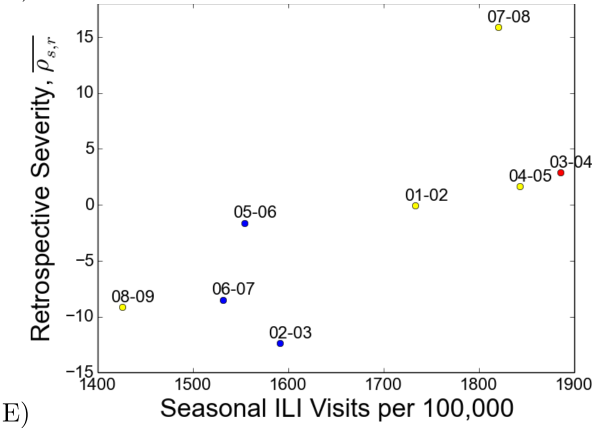

E)

Figure S10: Retrospective severity for the A) 2001-02 through 2008-09 medical claims data and the B) 1997-98 through 2013-14 (excluding 2009-10) ILINet data were compared to the proportion of H3 subtyped isolates during the flu season (NREVSS) (Pearson's R = 0.46 and 0.08, respectively). While non-H3 seasons were indicated with milder index values, H1 seasons also tend to have higher vaccine match in our study period, perhaps due to slower mutation rates or higher prevalence and better characterization during this period. The mechanistic relationship between severity and H3 proportion is thus confounded with the level of vaccine match. Nevertheless, B) suggested that our index captures both A/H1 and A/H3 seasonal flu severity. Point color corresponds to qualitatively-assigned severity category, where red is severe, yellow is moderate, and blue is mild.

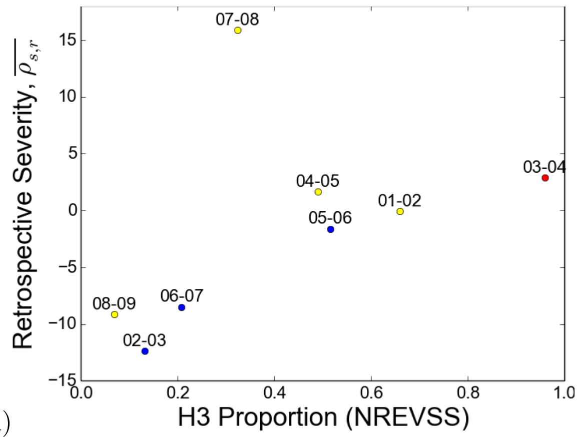

A)

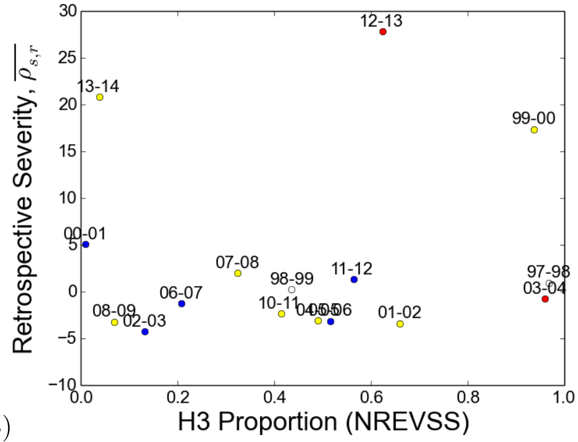

B)

Figure S11: A) Severity decreased as vaccine match increases (Pearson's R = -0.80). We clarify, however, that vaccine strain match tended to be higher for H1 subtypes, and H1-dominant seasons tend to be more mild. B) The relationship between severity and vaccine efficacy was uncertain. Vaccine efficacy percentages were estimated as a weighted average of study size in trivalent (TIV) and live-attenuated influenza vaccine (LAIV) efficacy trials. Point color corresponds to qualitatively-assigned severity category, where red is severe, yellow is moderate, and blue is mild.

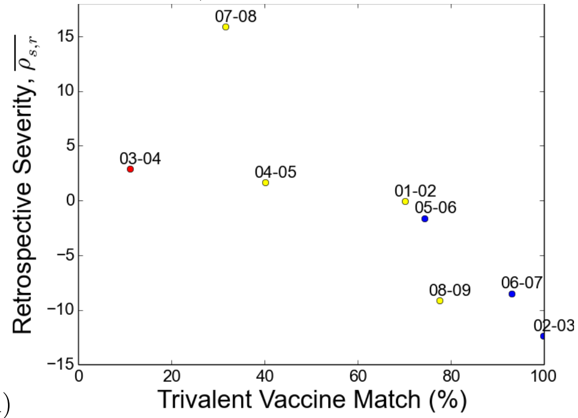

A)

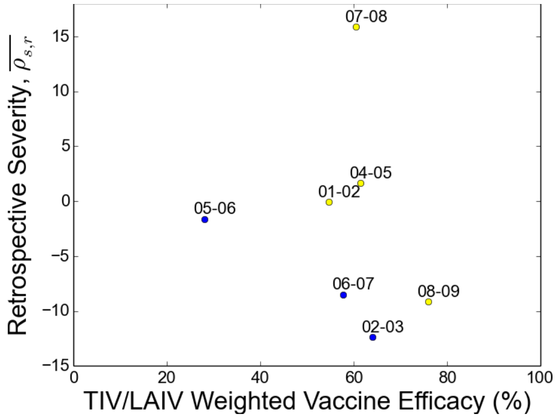

B)

Figure S12: Distribution of retrospective severity indexes by state, sorted by median severity value. Western states seemed to experience the mildest seasons. As with Figure 4b, the 75th and 70th percentiles of deviation between state and national retrospective severity exceeded zero for red and orange highlighted states, respectively. Missing states did not have indexes across all eight seasons.

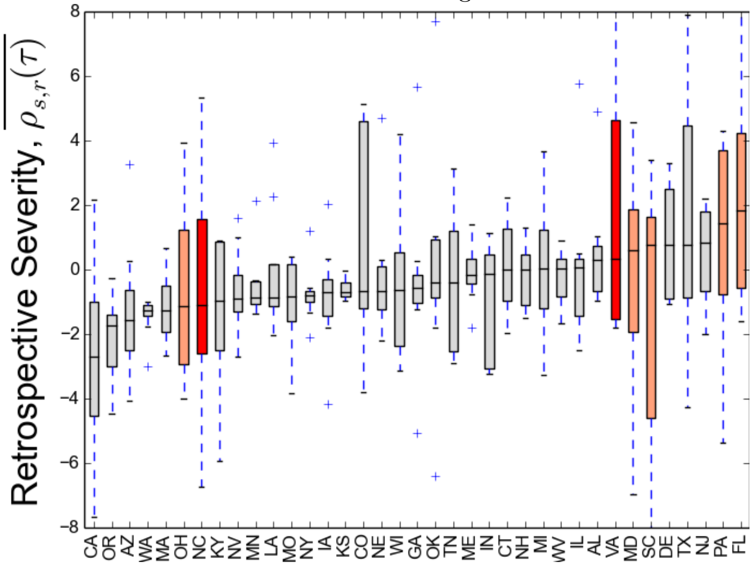

Figure S13: A) Medically attended ILI visits captured by ILINet over the 1997-98 to 2013-14 flu seasons (excluding 2009-10), adjusted for both increasing coverage of ILINet over time and health care seeking behavior for ILI. B) Standardized relative risk of ILI visits among adults to children ($\rho_{s,r}^{cdc}(t)$). C) Retrospective severity had a positive relationship with the benchmark (R = 0.64, p-value = 0.01). D) Early warning severity provided little information about severity (R = -0.17, p-value = 0.59). Point color in C & D corresponds to qualitatively-assigned severity category, where red is severe, yellow is moderate, blue is mild, and grey had no data.

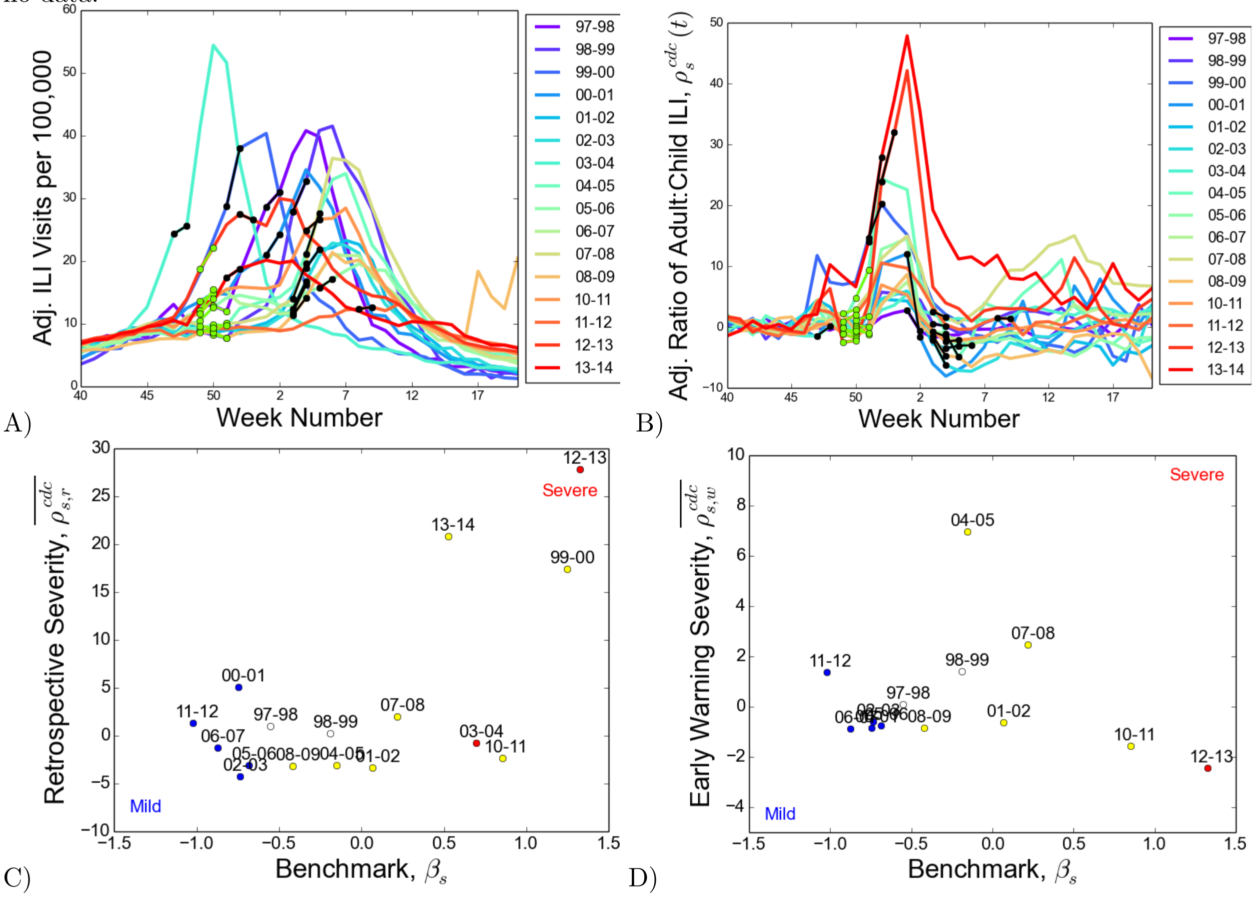

Figure S14: Comparison of severity indexes derived from the medical claims and ILINet data, for the A) retrospective and B) early warning indexes for the 2001-09 period. CDC data had a tendency to underestimate the severity index value as compared to the SDI data, but the retrospective classifications for both were highly correlated (Pearson's R = 0.78, p-value = 0.02). Early warning classifications graphically appeared to have a non-linear relationship. Point color corresponds to qualitatively-assigned severity category, where red is severe, yellow is moderate, and blue is mild. Grey diagonal line represents y = x.

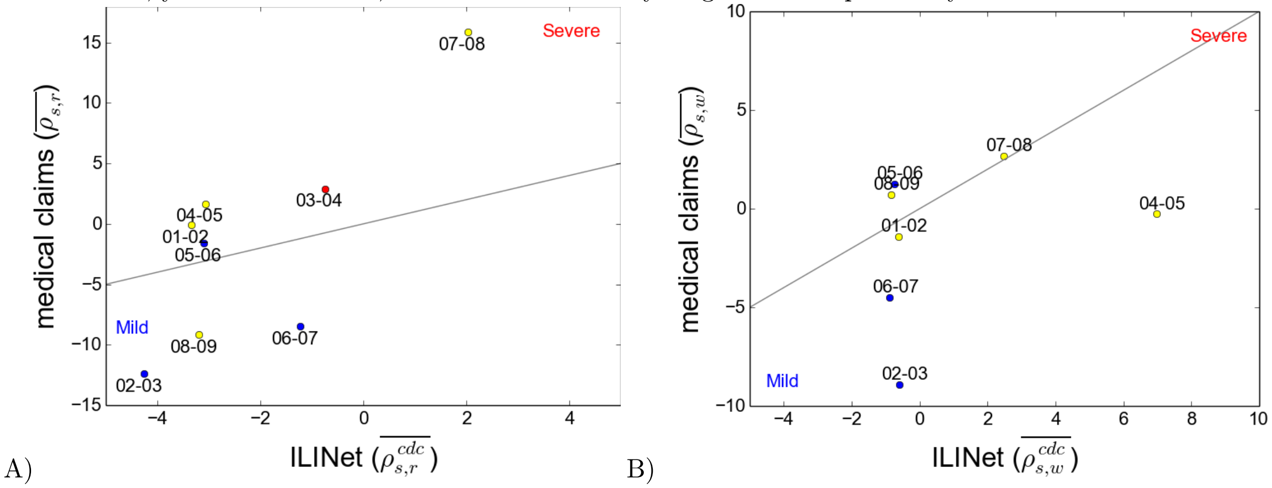

References

[1] Biggerstaff, M., Jhung, M., Kamimoto, L., Balluz, L., Finelli, L.: Self-reported influenza-like illness and receipt of influenza antiviral drugs during the 2009 pandemic, United States, 2009-2010. Am. J. Public Health **102**(10), 21–26 (2012). doi:10.2105/AJPH.2012.300651

[2] Biggerstaff, M., Jhung, M.A., Reed, C., Fry, A.M., Balluz, L., Finelli, L.: Influenza-like illness, the time to seek healthcare, and influenza antiviral receipt during the 2010-11 influenza season – United States. J. Infect. Dis. (2014)

[3] Jernigan, D.B., Lindstrom, S.L., Johnson, J.R., Miller, J.D., Hoelscher, M., Humes, R., Shively, R., Brammer, L., Burke, S.a., Villanueva, J.M., Balish, a., Uyeki, T., Mustaquim, D., Bishop, a., Handsfield, J.H., Astles, R., Xu, X., Klimov, a.I., Cox, N.J., Shaw, M.W.: Detecting 2009 pandemic influenza A (H1N1) virus infection: availability of diagnostic testing led to rapid pandemic response. Clin. Infect. Dis. **52 Suppl 1**(Suppl 1), 36–43 (2011). doi:10.1093/cid/ciq020

[4] Smith, D.J., Lapedes, A.S., de Jong, J.C., Bestebroer, T.M., Rimmelzwaan, G.F., Osterhaus, A.D.M.E., Fouchier, R.a.M.: Mapping the antigenic and genetic evolution of influenza virus. Science **305**(5682), 371–6 (2004). doi:10.1126/science.1097211

[5] Simonsen, L., Clarke, M.J., Williamson, G.D., Stroup, D.F., Arden, N.H., Schonberger, L.B.: The impact of influenza epidemics on mortality: introducing a severity index. Am. J. Public Health **87**(12), 1944–50 (1997)

[6] Lowen, A.C., Mubareka, S., Steel, J., Palese, P.: Influenza virus transmission is dependent on relative humidity and temperature. PLoS Pathog. **3**(10), 1470–6 (2007). doi:10.1371/journal.ppat.0030151

[7] Steel, J., Palese, P., Lowen, A.C.: Transmission of a 2009 pandemic influenza virus shows a sensitivity to temperature and humidity similar to that of an H3N2 seasonal strain. J. Virol. **85**(3), 1400–2 (2011). doi:10.1128/JVI.02186-10

[8] Longini, I.M., Koopman, J.S., Monto, a.S., Fox, J.P.: Estimating household and community transmission parameters for influenza. Am. J. Epidemiol. **115**(5), 736–51 (1982)

[9] Mossong, J., Hens, N., Jit, M., Beutels, P., Auranen, K., Mikolajczyk, R., Massari, M., Salmaso, S., Tomba, G.S., Wallinga, J., Heijne, J., Sadkowska-Todys, M., Rosinska, M., Edmunds, W.J.: Social contacts and mixing patterns relevant to the spread of infectious diseases. PLoS Med. **5**(3), 74 (2008). doi:10.1371/journal.pmed.0050074

[10] Longini, I.M., Halloran, M.E.: Strategy for Distribution of Influenza Vaccine to High-Risk Groups and Children. Am. J. Epidemiol. **161**(4), 303–306 (2005)

[11] Van Kerckhove, K., Hens, N., Edmunds, W.J., Eames, K.T.D.: The impact of illness on social networks: implications for transmission and control of influenza. Am. J. Epidemiol. **178**(11), 1655–62 (2013). doi:10.1093/aje/kwt196

[12] Wei, T.: corrplot: Visualization of a correlation matrix (2013). http://cran.r-project.org/package=corrplot

[13] Osterholm, M.T., Kelley, N.S., Sommer, A., Belongia, E.a.: Efficacy and effectiveness of influenza vaccines: a systematic review and meta-analysis. Lancet Infect. Dis. **12**(1), 36–44 (2012). doi:10.1016/S1473-3099(11)70295-X

[14] Viboud, C., Grais, R.F., Lafont, B.a.P., Miller, M.a., Simonsen, L.: Multinational impact of the 1968 Hong Kong influenza pandemic: evidence for a smoldering pandemic. J. Infect. Dis. **192**, 233–248 (2005). doi:10.1086/431150

[15] Reichert, T.a., Simonsen, L., Sharma, A., Pardo, S.a., Fedson, D.S., Miller, M.a.: Influenza and the winter increase in mortality in the United States, 1959-1999. Am. J. Epidemiol. **160**(5), 492–502 (2004). doi:10.1093/aje/kwh227
